# A Comprehensive Evaluation of the Relationship Between Different IgG and IgA Anti-Modified Protein Autoantibodies in Rheumatoid Arthritis

**DOI:** 10.3389/fimmu.2021.627986

**Published:** 2021-05-20

**Authors:** Caroline Grönwall, Lisa Liljefors, Holger Bang, Aase H. Hensvold, Monika Hansson, Linda Mathsson-Alm, Lena Israelsson, Vijay Joshua, Anna Svärd, Ragnhild Stålesen, Philip J. Titcombe, Johanna Steen, Luca Piccoli, Natalia Sherina, Cyril Clavel, Elisabet Svenungsson, Iva Gunnarsson, Saedis Saevarsdottir, Alf Kastbom, Guy Serre, Lars Alfredsson, Vivianne Malmström, Johan Rönnelid, Anca I. Catrina, Karin Lundberg, Lars Klareskog

**Affiliations:** ^1^ Department of Medicine Solna, Division of Rheumatology, Center for Molecular Medicine, Karolinska Institutet, Karolinska University Hospital, Stockholm, Sweden; ^2^ ORGENTEC Diagnostika GmbH, Mainz, Germany; ^3^ Center for Rheumatology, Academic Specialist Center, Stockholm Health Region, Stockholm, Sweden; ^4^ Rheumatology Clinic, Karolinska University Hospital, Stockholm, Sweden; ^5^ Thermo Fisher Scientific, Immuno Diagnostics Division, Uppsala, Sweden; ^6^ Department of Immunology, Genetics and Pathology, Uppsala University, Uppsala, Sweden; ^7^ Department of Biomedical and Clinical Sciences, Linköping University, Linköping, Sweden; ^8^ Center for Clinical Research Dalarna, Uppsala University, Uppsala, Sweden; ^9^ The Center for Immunology and Division of Rheumatic and Autoimmune Diseases, University of Minnesota Medical School, Minneapolis, MN, United States; ^10^ Institute for Research in Biomedicine, Università della Svizzera italiana, Bellinzona, Switzerland; ^11^ Unité Différenciation Épithéliale et Autoimmunité Rhumatoïde, INSERM - Université de Toulouse, Toulouse, France; ^12^ Department of Medicine Solna, Division of Clinical Epidemiology, Karolinska Institutet, Stockholm, Sweden; ^13^ Faculty of Medicine, School of Health Sciences, University of Iceland, Reykjavik, Iceland; ^14^ Institute of Environmental Medicine, Karolinska Institutet, Stockholm, Sweden; ^15^ Centre for Occupational and Environmental Medicine, Stockholm Health Region, Stockholm, Sweden

**Keywords:** autoantibodies, anti-citrullinated protein antibodies (ACPAs), anti-modified protein antibodies, anti-CCP antibodies, rheumatoid arthritis, anti-carbamylated protein antibodies, anti-acetylated protein antibodies

## Abstract

Seropositive rheumatoid arthritis (RA) is characterized by the presence of rheumatoid factor (RF) and anti-citrullinated protein autoantibodies (ACPA) with different fine-specificities. Yet, other serum anti-modified protein autoantibodies (AMPA), e.g. anti-carbamylated (Carb), -acetylated (KAc), and malondialdehyde acetaldehyde (MAA) modified protein antibodies, have been described. In this comprehensive study, we analyze 30 different IgG and IgA AMPA reactivities to Cit, Carb, KAc, and MAA antigens detected by ELISA and autoantigen arrays in N=1985 newly diagnosed RA patients. Association with patient characteristics such as smoking and disease activity were explored. Carb and KAc reactivities by different assays were primarily seen in patients also positive for anti-citrulline reactivity. Modified vimentin (mod-Vim) peptides were used for direct comparison of different AMPA reactivities, revealing that IgA AMPA recognizing mod-Vim was mainly detected in subsets of patients with high IgG anti-Cit-Vim levels and a history of smoking. IgG reactivity to acetylation was mainly detected in a subset of patients with Cit and Carb reactivity. Anti-acetylated histone reactivity was RA-specific and associated with high anti-CCP2 IgG levels, multiple ACPA fine-specificities, and smoking status. This reactivity was also found to be present in CCP2+ RA-risk individuals without arthritis. Our data further demonstrate that IgG autoreactivity to MAA was increased in RA compared to controls with highest levels in CCP2+ RA, but was not RA-specific, and showed low correlation with other AMPA. Anti-MAA was instead associated with disease activity and was not significantly increased in CCP2+ individuals at risk of RA. Notably, RA patients could be subdivided into four different subsets based on their AMPA IgG and IgA reactivity profiles. Our serology results were complemented by screening of monoclonal antibodies derived from single B cells from RA patients for the same antigens as the RA cohort. Certain CCP2+ clones had Carb or Carb+KAc+ multireactivity, while such reactivities were not found in CCP2- clones. We conclude that autoantibodies exhibiting different patterns of ACPA fine-specificities as well as Carb and KAc reactivity are present in RA and may be derived from multireactive B-cell clones. Carb and KAc could be considered reactivities within the “Cit-umbrella” similar to ACPA fine-specificities, while MAA reactivity is distinctly different.

## Introduction

Rheumatoid arthritis (RA) can be classified as seropositive or seronegative based on the presence of anti-citrullinated protein autoantibodies (ACPA) and/or rheumatoid factor (anti-IgG Fc) (1). The seropositive subset, comprising approximately 70% of patients have a different etiology compared to seronegative RA, with *HLA-DRB1* shared epitope (SE) and smoking identified as risk factors (2, 3). ACPA, commonly measured with the anti-cyclic-citrullinated peptide 2 (CCP2) tests, are RA-specific autoantibodies and recent functional studies have indicated that they are involved in the causation of RA-associated symptoms (4–8). Interestingly, ACPA have been demonstrated to bind to a large number of citrullinated proteins including filaggrin, vimentin, fibrinogen, α-enolase, and histones (9–12), and different patients express distinct profiles of these ACPA fine-specificities (13, 14).

Citrullination is a conversion of peptidyl-arginine into peptidyl-citrulline, mediated by peptidyl arginine deiminase (PAD) enzymes, which occurs during physiological conditions but is increased during inflammation in various tissues, including the RA synovium (15–18). Moreover, a spectrum of other anti-modified protein autoantibody (AMPA) reactivities to post-translational modifications (PTMs), such as carbamylated (Carb), acetylated (KAc) and malondialdehyde-modified (MDA) proteins, have been described in RA patients (19–24). In contrast to citrullination, which modifies arginine, the chemical modification carbamylation and the enzyme-regulated acetylation both result in lysine derivatives. The reactive aldehyde MDA, can mediate a range888 of amino acid modifications but the ring-formed lysine modification DHP-lysine, which is a malondialdehyde acetaldehyde (MAA) adduct generated in the presence of acetaldehyde, has been suggested to be particularly targeted by autoantibodies (25).

Recent studies using monoclonal antibodies have revealed that the ACPA serology profiles do not necessarily reflect parallel evolution of many different Cit-reactive clones but instead individual B-cell/antibody clones most often display multi-reactivity to a range of Cit-proteins (26–30). These clones have distinct selectivity that is explained by recognition of different small citrulline-containing peptide epitopes that can occur in several proteins (26). In addition, around 50% of Cit-reactive clones can also bind to carbamylated proteins and 25-30% of them bind acetylated antigens (26, 27, 31, 32). Still, the evolution and impact of these unique autoimmune multireactivity profiles in the etiology and pathogenesis of RA remains elusive.

In the current study we explore autoreactivity to different PTMs in individuals at risk of developing RA and early RA patients, and provide a comprehensive summary of how different IgG and IgA autoreactivities are related and how the multireactivity profiles can define patient subsets. The panel also includes the less studied reactivities to malondialdehyde acetaldehyde and acetylated histone that are here assessed in both RA and at-risk RA individuals. The association of AMPA IgG and IgA reactivity to classical RA risk factors, i.e. smoking and HLA-DRB1 SE alleles, as well as disease activity at first rheumatology visit are investigated. By using multiple autoantibody screening platforms we can determine complete AMPA profiles for the patients.

## Materials and Methods

### Clinical Samples

Autoantibodies were screened in serum samples from newly diagnosed RA patients with disease duration less than one year and population-based controls from the Epidemiological Investigation of Rheumatoid Arthritis (EIRA) cohort (33). In total 1985 RA patients and 480 controls were included in the study but not all samples were available for all biomarker assays. Inclusion was based on the 1987 ACR RA classification criteria (34), and the CCP2 autoantibody status was determined using the anti-CCP2 assay (CCPlus, Euro Diagnostica) with a saturation value at 3200 AU/ml. Serum samples were obtained at inclusion in the EIRA study. For evaluation of disease activity at diagnosis and six months clinical follow up, the EIRA cohort was linked to the electronic Swedish Rheumatology Quality register (SRQ) (35). Disease activity was assessed with 28 joint score (DAS28) including erythrocyte sedimentation rate (ESR) or C Reactive Protein (CRP). Patients were genotyped for *HLA-DRB1* alleles as previously described (3), and patients were determined to be shared epitope positive if carrying HLA-DRB1*01 (except *0103), *04 or *10. Smoking status was collected through questionnaires and individuals were categorized as “ever smokers” (including current and former smokers) or “never smokers”. IgM, IgG, and IgA rheumatoid factor (RF) analysis was performed using EliA immunoassays with a Phadia 2500 instrument (Phadia AB) according to the manufacturer’s instructions. IgG anti-MAA and IgG anti-KAc-His2B autoantibody levels were also assessed in 267 RA-risk individuals. The RA-risk individuals were IgG anti-CCP2 positive individuals referred to rheumatologist due to musculoskeletal symptoms suspicious for rheumatic disease, with no signs of inflammatory arthritis at joint examination and by ultrasound evaluation. For assay development, 159 SLE patients from the cross-sectional Karolinska SLE cohort were used as disease controls (36). The SLE patients fulfilled at least four of the 1982 revised ACR classification criteria (37). Disease activity was measured by the SLE disease activity index 2000 (SLEDAI-2K) (38). SLE-associated autoantibody levels (dsDNA, SSA/Ro52, SSA/Ro60, SSB/La, Sm, RNP, nucleosome, ribosomal-P antigen, cardiolipin and β2-glycoprotein-I) were available from multiplexed bead assays using BioPlex 2200 system (Bio-Rad). In line with Swedish law, patient consent was documented in the medical records by respective treating physician. This was done after the patient had received information about the study and after approving participation in the study (consent). The study was approved by the regional ethics review board in Stockholm.

### AMPA Screening

Presence of serum IgG ACPA fine-specificities were screened using a custom-made multiplex solid phase microarray platform (Thermo Fisher Scientific, ImmunoDiagnostics) (13) as previously reported (39). Sequences from the ten citrullinated peptides included in the analysis are presented in **Supplemental Table 1**. In peptides with multiple citrulline sites, the peptides were classified in the “Cit-Gly” group if any of the sites had a Cit-Gly motif. The array also included a previously described Carb-CEP1 peptide with the two arginine/citrulline residues in the autocyclic α-enolase peptide exchanged for homocitrulline-lysine (40).

Reactivity to acetylated histone 4 (KAc5; KAc16; or KAc-His4 (1–18) with multiple KAc) and histone 2B (KAc12 autocyclic) peptides were screened using in-house developed ELISAs with previously identified peptides (32) (sequences are available in **Supplemental Table 1**). Briefly, biotinylated peptides were captured on pre-coated streptavidin high capacity plates (Thermo Fisher Scientific) in 1% BSA in PBS. Serum samples were analyzed at 1:200 dilution in RIA buffer (1% BSA, 325 mM NaCl, 10 mM Tris-HCl, 1% Tween-20, 0.1% SDS), incubated 1.5 h, plates were washed, and reactivity was detected using HRP goat F(ab’)_2_ anti-human IgG γ (Jackson ImmunoResearch) followed by development with TMB substrate (Biolegend). The reactivity was quantified using standard curves of appropriate previously published single B-cell derived human monoclonal AMPA IgG1 from RA patients [1325:01B09 (27) or 37CEPT1G09 (41)] and Arbitrary Units (AU) per ml extrapolated. Equivalent native, lysine containing, peptides were run in parallel on the same plates and AU/ml values were corrected by subtraction. Serum IgG reactivity at 1:200 dilution to previously identified carbamylated fibrinogen peptides (Carb-Fibβ 43-56 and Carb-Fibβ 77-87) (42) were similarly analyzed using biotinylated peptides. Cut-off for positivity for ΔKAc-lys was set as mean value plus three standard deviations (SD) for 317 EIRA population controls.

IgG anti-malondialdehyde acetaldehyde protein levels were determined by an optimized ELISA protocol based on previously reported methodology (19, 43). Briefly, MAA bovine serum albumin (BSA) was generated by 2 h 37°C incubation of BSA (New England Biolabs) with 100 mM MDA (with acid activation of 1,1,3,3 tetrametoxypropane) in PBS and 50 mM acetaldehyde followed with extensive buffer exchange by dialysis to PBS (slide-a-lyzer 10K, Thermo Fisher Scientific). High-binding half area ELISA plates (Corning) were coated with 3 µg/ml MAA-BSA in PBS overnight, blocked with 1% casein in PBS, and reactivity was assessed in serum samples at 1:200 dilution in 1% BSA 0.1% casein in PBS. Reactivity was detected using HRP goat F(ab’)_2_ anti-human IgG γ and quantified using a previously published single-cell derived human monoclonal anti-MDA/MAA IgG1 isolated from RA synovium [1276:01F04 (19)] as standard curve with AU/ml values extrapolated. Since anti-MAA can be present to some extent in population controls [consistent with our previous study (43)], we used a cutoff for elevated/high IgG anti-MAA, that was set to the 85^th^ percentile of 317 EIRA population controls.

AMPA serum levels in RA patients were also assessed using the Orgentec modified vimentin (mod-Vim) ELISA system. The assay is based a 12 amino acid vimentin sequence (GRVYATRSSAVR) derived from the vimentin protein used in the MCV assay (20). It utilizes biotinylated peptides of identical length and composition that differ at one amino acid residue. The original arginine/citrulline site in the modified vimentin peptide is replaced by lysine (Lys), ornithine (Orn), homocitrulline (Carb), acetyl-ornithine [Orn(Ac)] or acetyl-lysine [Lys(Ac)] as previously reported (23).

IgG and IgA peptide assays were performed in parallel at Orgentec Diagnostika and reported as OD values. For the IgG assays we used the 97^th^ percentile among 480 EIRA controls for cutoff for positivity. Due to the difference in distribution, the cutoff had to be set to the double OD for the 97^th^ percentile for IgA to avoid false positivity.

Furthermore, screening of IgG reactivity to carbamylated full-length fibrinogen and carbamylated fetal calf serum (FSC) has previously been reported (24). Cutoff for positivity was set based on average reactivity + 2×SD in 316 EIRA population controls. Similarly, screening of IgA anti-CCP2 levels has previously been published and was assessed by EliA™ (Phadia AB) with a Phadia 250 instrument (Phadia AB) (44). IgA anti-CCP2 cutoff was set to 2 µg/ml based on analysis in Svärd et al. (44). Total serum IgA levels were measured by reversed-phase microarray methodology and total IgM levels were assessed by ELISA as previously reported (45, 46).

### RA-Derived Monoclonal Antibodies

Generation of human monoclonal antibodies from BCR sequences from single cell sorted RA B-cells was achieved following our previously published protocol (47). Briefly, BCR variable region transcripts were PCR amplified, cloned into heavy and light chain expression vectors and recombinant antibodies were expressed as IgG1 in Expi293 cells (Thermo Fisher Scientific), purified, and subjected to quality controls. The 16 anti-CCP2 ACPA clones used here (27, 41, 48) have previously been identified and extensively evaluated for Cit-peptide reactivity by positivity at 5 µg/ml in CCP2 ELISA, Cit-peptide microarray screening and Cit-peptide ELISA (26, 27, 32, 41, 49). None of CCP2+ clones had any native peptide reactivity. In total 250 non-ACPA mAb clones obtained from different RA patients and compartments were expressed as IgG1 and screened together with the ACPA clones for reactivity to different modified peptides at 5 µg/ml IgG following the same protocols as above. We excluded clones positive in a polyreactivity assay utilizing the soluble membrane protein (SMP) fraction from Hek293 cells for evaluation of unspecific binding (26).

### Statistical Methods

Differences in autoantibody levels between groups were evaluated with Mann-Whitney or Kruskal-Wallis analysis with Dunn’s correction for multiple comparisons. Differences in frequencies of positive tests were assessed with Fisher’s exact test. Unsupervised hierarchical clustering was performed using the Ward method. Spearman analysis or Kendell analysis was performed for direct correlations of continuous values. For analysis adjusting for co-variables (e.g. sex, age, CCP2 levels) we used logistic regression. Heatmap visualization of categorical values in seronegative RA was performed using Cluster 3.0 and Java TreeView. Statistical analysis was performed using JMP 14 (SAS institute) and Prism 6 (Graphpad) and p-values < 0.05 were considered statistically significant.

## Results

### ACPA Fine-Specificity and RF Isotype Reactivity Capture Additional Seropositive Patients Compared to Routine Anti-CCP and RF IgM Assays

Recent advances in RA serology have suggested that the IgG anti-CCP and RF tests are not capturing all autoreactivities in RA, and that a subset of patients can also display a number of anti-modified protein autoantibody reactivities besides ACPA. In the current investigation we therefore took advantage of multiple antibody detection systems to generate an as complete as possible picture of RA autoantibodies in our early RA cohort (**Table 1** and **Supplemental Table 1**).

**Table 1:** AMPA reactivities in RA patients.

	Controls	SLE	RA-Risk	All RA	CCP-RA	CCP+ RA
***AMPA frequencies***						
CCP2 IgA	N/A	N/A	N/A	45% (882/1942)	8% (59/729)	68% (823/1213)
Δac-lys His2B [>4.25 AU/ml]*	0% (0/437)	3.8% (6/160)	12.7% (34/267)	17% (68/403)	2.4% (3/125)	23% (65/278)
mod-Vim IgG Cit [OD>0.55]#	2.9% (14/480)	N/A	N/A	60% (1188/1982)	11% (84/733)	88% (1104/1249)
mod-Vim IgG Orn(Ac) [OD>0.55]#	2.7% (13/480)	N/A	N/A	51% (1007/1982)	7.6% (56/733)	76% (951/1249)
mod-Vim IgG Carb [OD>0.69]#	2.7% (13/480)	N/A	N/A	41% (809/1982)	6% (44/733)	61% (765/1249)
mod-VimIgG Lys(Ac) [OD>0.68]#	2.9% (14/480)	N/A	N/A	29% (571/1982)	4.4% (32/733)	43% (539/1249)
mod-Vim IgA Cit [OD>0.32]€	0.6% (3/480)	N/A	N/A	17% (333/1982)	1.2% (9/733)	26% (324/1249)
mod-Vim IgA Orn(Ac) [OD>0.36]€	0.6% (3/480)	N/A	N/A	10% (199/1982)	2.2% (16/733)	15% (183/1249)
mod-Vim IgA Carb [OD>0.48]€	0% (0/480)	N/A	N/A	5.8% (115/1982)	2.9% (21/733)	7.5% 94/1249)
mod-Vim IgA Lys(Ac) [OD>0.36]€	0.2% (1/480)	N/A	N/A	7.5% (149/1982)	1.9% (14/733)	11% (135/1249)
Carb-Fib IgG [>23 AU/ml]§	2.5% (8/316)	N/A	N/A	43% (844/1982)	12% (90/730)	60% (754/1255)
Carb-FCS IgG [>232 AU/ml]§	3.1% (10/316)	N/A	N/A	36% (705/1982)	12% (85/730)	49% (621/1255)
MAA IgG [high >56.44 AU/ml]**	14% (61/437)	44% (70/159)	13% (39/267)	44% (176/403)	33% (41/125)	49% (135/278)
***AMPA levels mean±SD (median)***						
KAc-His2B IgG	1.4±3.1 (0.53)	5.2±9.4 (1.4)	5.0±7.7 (1.6)	4.4±9.0 (0.84)	1.2±2.4(0.46)	5.9±10 (1.5)
Δac-lys His2B IgG	-0.11±1.4 (0.01)	-1.2±4.2 (-0.25)	2.0±6.1 (0.20)	3.2±8.6 (0.10)	0.29±1.5 (-0.001)	4.5±10 (0.36)
mod-Vim IgG Cit	0.18±0.31 (0.08)	N/A	N/A	1.6±1.4 (1.3)	0.33±0.57 (0.17)	2.4±1.2 (2.7)
mod-Vim IgG Orn(Ac)	0.21±0.26 (0.15)	N/A	N/A	1.2±1.1 (0.57)	0.29±0.24 (0.21)	1.6±1.1 (1.5)
mod-Vim IgG Carb	0.19±0.26 (0.13)	N/A	N/A	0.89±0.93 (0.49)	0.28±0.31 (0.20)	1.3±1.0 (0.95)
mod-Vim IgG Lys(Ac)	0.16±0.22 (0.11)	N/A	N/A	0.70±0.82 (0.35)	0.27±0.27 (0.20)	0.94±0.93 (0.55)
mod-Vim IgA Orn(Ac)	0.07±0.05 (0.05)	N/A	N/A	0.21±0.29 (0.14)	0.16±0.15 (0.14)	0.24±0.34 (0.14)
mod-Vim IgA Carb	0.07±0.05 (0.06)	N/A	N/A	0.19±0.22 (0.13)	0.16±0.17 (0.13)	0.20±0.24 (0.14)
mod-Vim IgA Lys(Ac)	0.07±0.05 (0.06)	N/A	N/A	0.18±0.24 (0.12)	0.13±0.14 (0.11)	0.21±0.27 (0.13)
Carb-Fib IgG	3.6±11 (0.31)	N/A	N/A	75±210 (17)	21±106 (4.7)	107±246 (33)
Carb-FCS IgG	56±140 (22)	N/A	N/A	311±448 (137)	114±218 (52)	425±504 (229)
MAA IgG	33.7±27 (26)	65±47 (48.3)	36±26 (27)	62±39 (49.8)	54±39 (43)	65±39 (55)

*Mean of 316 controls + 3SD; **85^th^ percentile of 317 controls; ^#^97^th^ percentile of 480 controls.

^§^Mean of 316 controls + 2SD; ^€^97^th^ percentile of 480 controls x2.

Values for control assays are available in **Supplemental Table 2**.

Comparable with previous studies (39, 50–52), we identified more ACPA+ patients using the ACPA fine-specificity array than with the IgG CCP2 test alone, with around 10% (38/393) being ACPA+ CCP2-. And as previously shown (52), these patients with a negative CCP2 IgG test but positive fine-specificity test, displayed fewer ACPA fine-specificities compared to CCP2+ patients (Mean 2.1±1.9 of 10 Cit-peptides, *vs* to 5.9±2.5, p<0.0001). Similarly, around 3% of the patients who were negative in the anti-CCP2 IgG test were anti-CCP2 IgA positive (59/1942).

When including RF IgG and IgA tests, an additional 5% of the patients were shown to be RF positive compared to the results from the RF IgM alone (91/1985), which was similar to what was observed in our recent report (52). Altogether, we found that 24% (464/1942) of the patients were truly seronegative i.e. anti-CCP2 IgG/IgA- and RF IgM/IgG/IgA-. Citrulline reactivity by different methodologies and any RF isotype reactivity showed a significant overlap, yet around 10% (39/386) of the patients were RF positive (IgM, IgG, and/or IgA) without any detectable citrulline reactivity by CCP2 and multiplex ACPA fine-specificity assays (**Supplemental Figure 1**).

### Overlap Between Anti-Carb-Fibrinogen/FCS Assays and Anti-CCP2

Our cohort data set also included previously reported measurements of IgG reactivity to carbamylated fibrinogen and carbamylated FCS (**Supplemental Figure 2** and **Table 1**) (24). When combining results from the two assays, 70% of CCP2+ patients were also positive for IgG anti-Carb (891/1250). The levels and frequency of anti-Carb positive patients were significantly lower in CCP2- RA patients. Nevertheless, 21% of CCP2- (157/735) were positive for IgG anti-Carb fibrinogen and/or anti-Carb-FCS, in line with our previous data, where we could also show that approximately 40% of the Carb-Fib+/CCP2-/IgM RF- patients were ACPA fine-specificity positive (24, 52). We also screened a smaller subset of controls and RA patients for IgG reactivity to two carbamylated fibrinogen β-chain peptides (Fibβ 43-56 and Fibβ 77-87). We observed significantly increased reactivity to these peptides in RA patients compared to controls (**Supplemental Figure 3**).

### Synthetic Peptide Motifs Reveal the Level of AMPA Reactivity in RA

**Figure 1:**
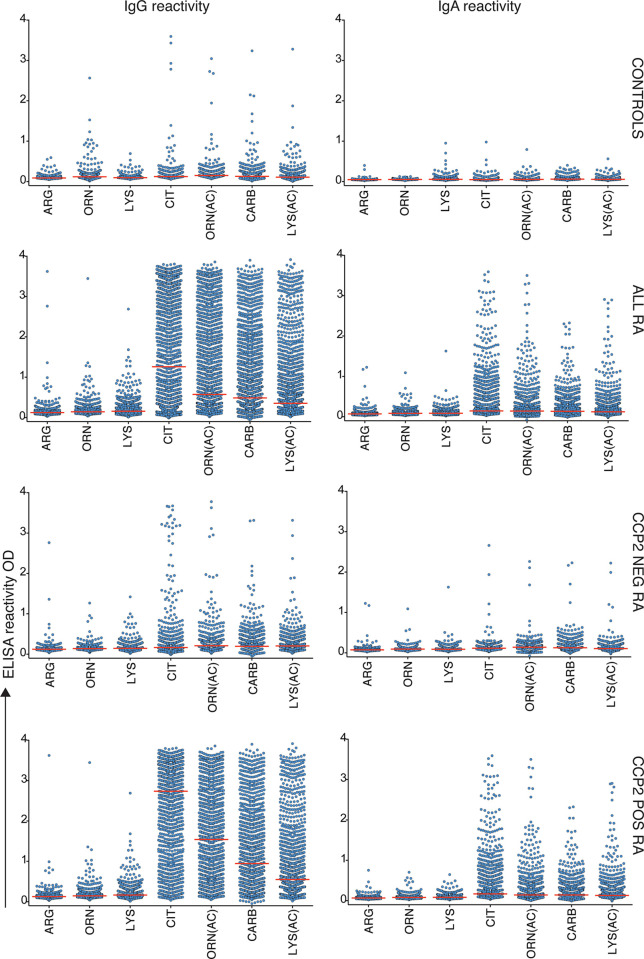
AMPA reactivity in early RA by the modified vimentin assay. Results from mod-Vim ELISA screening in 480 population controls and 1984 RA patients whereof 733 were ACPA negative by the CCP2 assay and 1249 were CCP2 positive. All antibody levels (Arg, Orn, Lys, Cit, Orn(Ac), Carb and Lys(Ac)) were significantly higher (p < 0.0001) in all RA patients, CCP2- RA, and CCP2+ RA, compared to the population controls using Kruskal-Wallis test with Dunn’s correction for multiple comparisons. The mod-Vim assay is based on the modified Vim58-69 peptide. Red lines depict medians.

To be able to directly compare reactivity to different post-translational modifications, controlling for the influence of protein backbone, we used the mod-Vim assay system for detection of reactivity to Cit, Carb, Lys(Ac) and Orn(Ac). These assays are using the same peptide derived from human vimentin (Vim) and replacing the original arginine with the modified amino acid residue. The results demonstrate that while IgG anti-Cit-Vim provided the highest reactivity in RA, patients also had substantial reactivity to the other modified Vim-peptides (**Table 1** and **Figure 1**) whereas the population controls displayed none or very limited reactivity in the assays. Notably, in comparison, the reactivity was low to the control peptides containing arginine, lysine or ornithine (**Supplemental Table 2** and **Figure 1**). A vast majority of the patients with strong IgG anti-modified peptide (Carb, Lys(Ac), Orn(Ac)) reactivity were CCP2 positive and the frequency of mod-Vim positivity was significantly higher in CCP2+ RA compared to CCP2- RA (Cit 88% *vs.* 11%; Orn(Ac) 76% *vs*. 7.6%; Carb 61% *vs*. 6%; Lys(Ac) 43% *vs*. 4.4%). However, statistically, antibody levels measured with these assays were also significantly increased in CCP2- RA compared to controls. IgA reactivity to the modified peptides was also increased in CCP2+ RA as discussed in more detail below.

**Figure 2:**
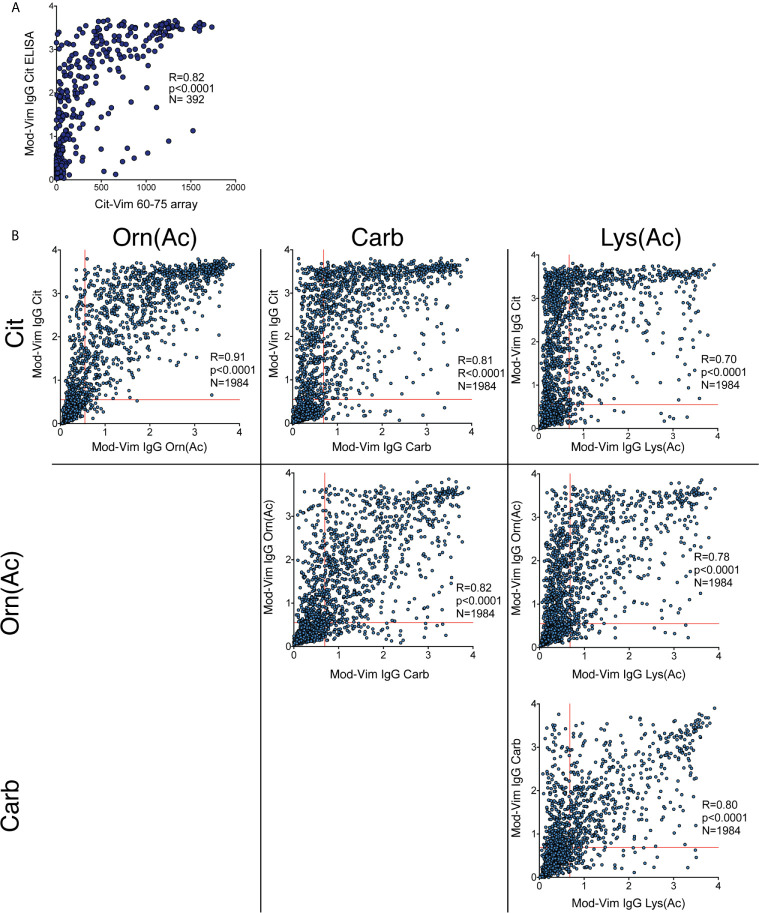
Correlation between different IgG AMPA reactivities by the modified vimentin assay. **(A)** Correlation of the IgG mod-Vim Cit-peptide ELISA based on the modified Vim58-69 peptide and Cit-Vim60-75 reactivity on the ACPA fine-specificity array. **(B)** Spearman correlation between the different modified peptide assays. Carb and Lys(Ac) reactivity was primarily seen in a subset of patients with high Cit-reactivity. Lys(Ac) reactivity was detected in a subset of patients with Carb-reactivity. Cutoff for positivity was determined by the 97^th^ percentile of the population controls.

The mod-Vim backbone peptide overlaps with the Cit-Vim60-75 peptide on the ACPA fine-specificity array, and we could confirm that the two assays showed a strong correlation (**Figure 2A**). When analyzing the correlation between the signals for the mod-Vim peptide modifications, it is evident that IgG Carb and Lys(Ac) reactivity was primarily observed in patients with high Cit-reactivity (**Figure 2B**). Yet, not all patients with high citrulline signal also have Carb and Lys(Ac) reactivity. Especially anti-Lys(Ac) seems to be present only in a smaller subset of patients. Interestingly, reactivity to the Orn(Ac) modification, which is the modification most structurally similar to citrulline (**Supplemental Figure 4**), showed a more direct correlation pattern with the anti-Cit reactivity.

### RA Patients Have Autoantibodies to Acetylated Histones

**Figure 3:**
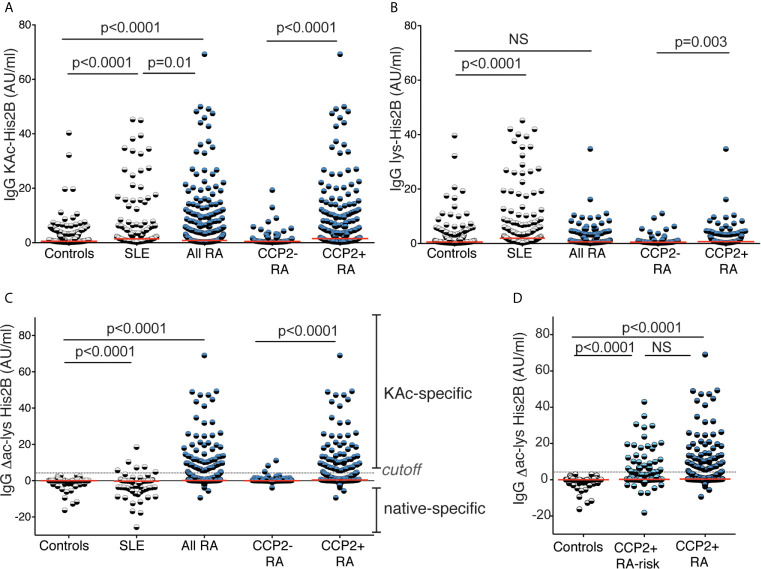
Reactivity to acetylated acetylated histone 2B. Serum IgG reactivity to acetylated histone 2B (K12) was determined by ELISA in 437 population controls, 160 SLE patients, 403 RA patients whereof 125 CCP2 negative and 278 CCP2 positive, and 267 CCP2 positive RA-risk individuals without arthritis. Reactivity to the acetylated (KAc) peptide **(A)** was compared to the native (lys) peptide **(B)**. **(C, D)** show acetylation reactivity normalized for native reactivity, Δac-lys His2B. Red lines depict medians. P-values are presented from Kruskal-Wallis test with Dunn’s correction for multiple comparisons. Positivity for Δac-lys His2B was set based on mean + 3SD for 316 population controls (4.25 AU/ml).

Acetylated histones represent interesting AMPA targets and we have previously identified several naturally occurring acetylation sites that can be recognized by RA-derived monoclonal ACPA (32). Here, for the first time, we investigated serum IgG levels to the acetyl-K12 site in histone 2B. We found that autoreactivity to KAc-His2B was significantly increased in RA patients compared to controls (**Figure 3**). Importantly, when analyzing SLE patients as disease controls, we found that they instead displayed reactivity to the native lysine-containing histone peptide, which was not seen in RA. This is consistent with anti-nuclear autoantibody and histone autoreactivity in SLE, and indeed the histone peptide reactivity correlated the strongest with nucleosome reactivity in the patients (**Supplemental Figure 5**). To control for native histone reactivity, all KAc-His2B values were normalized for lysine reactivity (Δac-lys) in further analysis of the RA cohort. The KAc-His2B reactivity was almost exclusively detected in CCP2+ RA, and made up 23% of this subset (**Tables 1** and **2**). However, it should be noted that two of the three IgG anti-KAc-His2B+ CCP2- individuals had detectable ACPA fine-specificities. We also had the opportunity to investigate lysine-acetylation and MAA reactivity in the risk-RA cohort (n=267). While the frequency of positivity for IgG anti-KAc-His2B was significantly lower in the risk-RA cohort compared to the CCP2+ early RA cohort (13% compared to 23%, p=0.001), the IgG anti-KAc-His2B levels were still significantly increased in RA-risk individuals compared to population controls (**Table 1** and **Figure 3D**).

**Table 2:** Baseline characteristics of CCP2+ RA patients with acetylated-histone reactivity.

	IgG KAc-His2B neg (n=213)	IgG KAc-His2B pos (n=65)		
	*Frequency % Mean±SD (Median)*	*Frequency % Mean±SD (Median)*	*OR[CI]*	p-values*
Age	49±23 (51)	51±11 (53)		NS (p=0.37)
Female	73% (156/213)	67% (44/65)	1.3 [0.72-2.4]	NS (p=0.43)
HLA shared epitope	88% (186/212)	81% (52/65)	0.6 [0.28-1.3]	NS (0.21)
HLA DRB1*01	25% (52/211)	27% (17/64)	1.1 [0.58-2.1)	NS (0.74)
HLA DRB1*04	69% (146/211)	69% (44/64)	0.98 [0.53-1.8]	NS (>0.99)
HLA DRB1*03	18% (39/211)	6% (4/64)	0.29 [0.10-0.85]	**p=0.02**
Ever smoking	70% (149/212)	86% (58/65)	2.6 [1.2-5.6]	**p=0.007**
DAS28	5.1±1.46 (5.3)	4.9±1.1 (5.1)		NS (p=0.31)
CRP	30.6±42.8(16.0)	23.9±21.7 (17)		NS (p=0.86)
IgG CCP2 levels (AU/ml)	803±861 (470)	1703±1096 (1417)		**p<0.0001**
IgG CCP2 high (>1000 AU/ml)	28% (59/213)	68% (44/65)	5.5 [3.0-10.0]	**p<0.0001**
IgA CCP2 pos	58% (123/211)	86% (52/60)	4.7 [2.1-10.3]	**p<0.0001**
IgA CCP2 levels (AU/ml)	6.7±10.5 (2.6)	13.6±12 (11)		**p<0.0001**
***ACPA IgG fine-specificities***				
Peptides with cit-gly motifs:				
Cit-Fil_307-324_ pos	53% (111/208)	87% (56/64)	6.1 [2.8-13.5]	**p<0.0001**
CEP-1 pos	59% (122/208)	84% (54/64)	3.8 [1.8-7.9]	**p=0.0002**
Cit-Fibβ_36-52_ pos	65%% (134/208)	90% (58/64)	5.3 [2.1-13]	**p<0.0001**
Cit-Fibα_563-583_ pos	58% (121/208)	84% (55/67)	3.9 [1.9-8.0]	**p<0.0001**
Cit-Fibα_580-600_ pos	28% (58/208)	64% (41/64)	4.6 [2.5-8.3]	**p<0.0001**
Cit-Fibα_621-635_ pos	38% (79/208)	71% (46/64)	4.1 [2-3-7.7]	**p<0.0001**
Peptides with other motifs:				
Cit-Vim_60-75_ pos	62% (130/208)	79% (51/64)	2.4 [1.2-4.6]	**p=0.01**
Cit-Vim_2-17_ pos	45% (93/208)	65% (42/64)	2.4 [1.3-4.2]	**p=0.004**
Cit-Fibα_36-50_ pos	22% (46/208)	14% (9/64)	0.6 [0.36-1.25]	NS (p=0.21)
Cit-Fibβ_60-74_ pos	80% (166/208)	92% (59/64)	3.0 [1.1-7.9]	**p=0.002**
Number of ACPA IgG fine-specificities (of 10)	5.1±2.7 (5)	7.3±2.2 (8)		**p<0.0001**
***AMPA IgG anti-***				
Carb-CEP1 pos	31% (64/208)	59% (38/64)	3.2 [1.8-5.9]	**p<0.0001**
mod-Vim Orn(Ac) pos	70% (150/212)	87% (57/65)	2.9 [1.3-6.5]	**0.005**
mod-Vim pos	57% (121/212)	58% (58/65)	6.2 [2.7-14]	**p<0.0001**
mod-Vim Lys(Ac) pos	33% (70/212)	73% (48/65)	5.7 [3.1-11]	**p<0.0001**
Carb-Fib pos	54% (114/212)	69% (45/65)	1.9 [1.1-3.5]	**p=0.03**
Carb-FCS pos	47% (100/212)	69% (45/65)	2.5 [1.4-4.6]	**p=0.002**
MAA high	49% (105/213)	49% (32/65)	1.0 [0.57-1.7]	NS (p>0.99)
***RF***				
IgG pos	70% (149/213)	75% (49/65)	1.3 [0.70-2.5]	NS (p=0.44)
IgA pos	48% (103/213)	49% (32/65)	1.0 [0.59-1.8]	NS (p>0.99)
IgM pos	84% (179/213)	80% (52/65)	0.8 [0.37-1.5]	NS (0.45)

*p-value from Fisher’s exact test or Mann-Whitney analysis.

OR, Odds ratio; CI, 95% confidence interval; NS, Not statistically significant. Bold font highlights significant p-values.

The characteristics of the IgG anti-KAc-His2B positive RA patient subset within the CCP2+ patients are presented in **Table 2**, and included an increased frequency of Cit-peptide positivity, high anti-CCP2 IgG and IgA levels, and a high frequency of Carb-reactivity. On the other hand, there was no significant difference in RF IgM/IgG/IgA positivity between anti-KAc-His2B IgG positive and negative patients. Neither was there any evidence for a more active disease or increased inflammation in the IgG anti-KAc-His2B positive subset by DAS28 or CRP observed compared to the anti-KAc-His2B negative anti-CCP2+ patients. We also noticed a higher frequency of ever smoking in the anti-KAc-His2B+ subset compared to the anti-KAc-His2B- CCP2+ subset. The association was confirmed with significantly higher IgG anti-KAc-His2B frequency in CCP2+ patients with a history of smoking compared to the other non-smoking CCP2+ patients (27% *vs.* 13%, p=0.01). Yet, the correlation did not reach statistical significance when adjusting for IgG anti-CCP2 levels (p=0.1) and may therefore not be considered independent (**Supplemental Table 3**).

While a majority of the IgG anti-KAc-His2B+ patients were HLA-DRB1 SE positive, there was no enrichment compared to the rest of the CCP2+ individuals (**Table 2** and **Supplemental Table 4**). Interestingly, there seems to be an inhibitory effect of HLA-DRB1*03 alleles on the occurrence of anti-KAc-His2B, as these alleles were significantly less frequent in anti-KAc-His2B+ individuals compared to anti-KAc-His2B- CCP2+ patient (**Table 2**). The anti-KAc-His2B levels were significantly higher in HLA-DRB1*03 negative also when stratifying for HLA shared epitope positivity (**Supplemental Table 5**). Notably, this association was significant even when adjusting for absence of two shared epitope alleles. Similar associations were seen for mod-Vim IgG anti-Lys(Ac) and IgG anti-Carb-FCS that were significantly higher in HLA-DRB1*03 negative and HLA shared epitope positive RA patients.

We also observed, in a smaller number of patients, that KAc-histone autoreactivity was not unique for this particular histone peptide, but RA-specific KAc-reactivity was also detected for three different acetylated Histone 4 peptides (KAc-His4[1-18] with multiple KAc sites or KAc5-His4; KAc16-His4 with single KAc sites; **Supplemental Figure 3**). These peptides covered different KAc-motifs (26) and although the reactivity correlated between the anti-acetylated histone assays, the patterns were slightly different for different patients.

### Autoreactivity to Malondialdehyde Acetaldehyde Adducts Is Increased in RA and SLE

**Figure 4:**
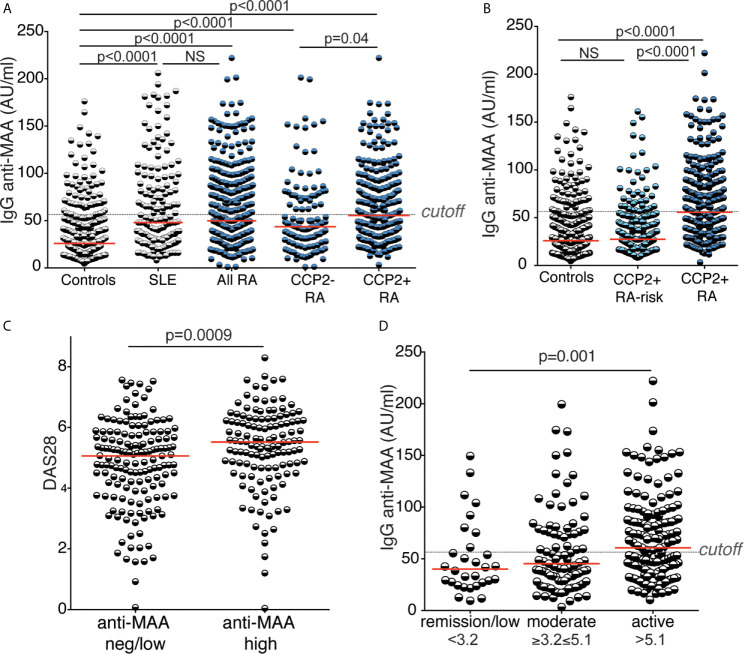
Reactivity to malondialdehyde acetaldehyde modified protein. Serum IgG reactivity to malondialdehyde acetaldehyde (MAA) modified protein was determined by MAA-BSA ELISA in 437 population controls, 159 SLE patients, 403 RA patients whereof 125 CCP2 negative and 278 CCP2 positive, and 267 CCP2 positive RA-risk individuals without arthritis. Cutoff for high reactivity was set to the 85^th^ percentile of 316 population controls (56.44 AU/ml). **(A, B)** MAA reactivity in different cohorts. **(C)** MAA reactivity in association with RA disease activity by DAS28 in 281 RA patients (153 with low MAA levels and 128 with high MAA levels, > 56.44 AU/ml). **(D)** IgG MAA reactivity in 30 RA patients with low disease activity (DAS28 < 3.2), 93 with moderate disease activity (3.2 ≤ DAS28 ≥ 5.1), and 158 with high disease activity (DAS28 > 5.1). All RA patients had early disease (< 1 year after symptom onset). Red lines depict medians. P-values are presented from Kruskal-Wallis test with Dunn’s correction for multiple comparisons.

An autoreactivity that has gained increasing interest is IgG anti-malondialdehyde and malondialdehyde acetaldehyde (MDA/MAA). We observed a significant increase in IgG to MAA-adducts in RA patients compared to controls by ELISA using MAA-modified bovine serum albumin (**Figure 4** and **Table 1**). IgG anti-MDA/MAA levels were detectable but significantly lower in the population controls, which is consistent with our previous investigations of IgG anti-MDA (43) and the hypothesis that this AMPA IgG is related to natural antibodies. Moreover, SLE patients displayed the same level of IgG anti-MAA as in RA, supporting previous data that anti-MAA is not RA-specific (43). Nevertheless, IgG anti-MAA was significantly elevated in CCP2+ RA compared to CCP2- RA (49% with high anti-MAA in CCP2+ compared to 33% in CCP2- RA and 14% in controls). Yet, the differences were less dramatic compared to other investigated autoreactivities and the frequency of individuals with elevated IgG anti-MAA above cutoff in the CCP2+ compared to CCP2- did not reach significance when adjusting for sex, age and DAS28 (p=0.19; data not shown). We observed an association with disease activity by DAS28, i.e. RA patients with more active disease had higher levels of IgG anti-MAA (**Figure 4** and **Table 3**; **Supplemental Figures 6, 7**). It should be noted that no other AMPA or RF isotype tests showed any correlation with disease activity in our analysis (**Supplemental Figure 6**). The only other antibody analysis result that correlated with anti-MAA was interestingly enough total IgA levels. In SLE IgG anti-MDA/MAA equivalently correlated with the SLEDAI score (**Supplemental Figure 5**). Moreover, in contrast to IgG anti-KAc-His2B, neither the average IgG anti-MAA levels nor the frequency high IgG anti-MAA above cutoff, were significantly elevated in the RA-risk cohort compared to population controls (**Table 1** and **Figure 4**). When breaking down the components of DAS28, we primarily observed a correlation with the inflammatory components (**Supplemental Table 6**). There was also an association of baseline anti-MAA with decreased ESR on 6-month follow up after DMARD treatment (**Supplemental Table 7**).

**Table 3:** Baseline characteristics of RA patients with high anti-MAA levels.

	IgG MAA low (n=227)	IgG MAA high# (n=176)		
	*Frequency % Mean±SD (Median)*	*Frequency % Mean±SD (Median)*	*OR [CI]*	p-values*
Age	50.7 ± 12.4 (53)	50.1 ± 12.8 (53)		NS (p=0.64)
Female	72% (163/227)	67% (118/176)	1.3 [0.82-1.9]	NS (p=0.33)
HLA Shared epitope	76% (172/225)	78% (134/172)	1.1 [0.66-1.7]	NS (p=0.81)
HLA DRB1*01	26% (59/224)	21% (36/173)	0.73 [0.46-1.2]	NS (p=0.23)
HLA DRB1*04	58% (129/224)	61% (106/173)	1.2 [0.78-1.75]	NS (p=0.47)
HLA DRB1*03	20% (45/224)	19% (33/173)	0.94 [0.57-1.5]	NS (p=0.90)
Smoking (present or previous)	70% (159/226)	73% (128/175)	1.1 [0.74-1.8]	NS (p=0.58)
DAS28	4.8 ± 1.4 (5.1)	5.3 ± 1.4 (5.5)		**p<0.001**
CRP	24.5 ± 37.4 (12)	33.4 ± 36.7 (18)		**p=0.0008**
IgG CCP2 levels (AU/ml)	627 ± 913 (137)	795 ± 989 (368)		**p=0.006**
IgG CCP2 pos	63% (143/227)	77% (135/176)	1.9 [1.2-3.0]	**p=0.003**
IgA CCP2 levels (AU/ml)	5.8 ± 9.9 (1.6)	6.7 ± 10.0 (2.2)		**p=0.005**
IgA CCP2 pos	43% (96/223)	53% (92/172)	1.5 [1.1-2.3]	**p=0.04**
***Cit IgG fine-specificities***				
Peptides with cit-gly motifs:				
Cit-Fil_307-324_ pos	41% (90/221)	47% (82/173)	1.3 [0.87-1.96]	NS (p=0.22)
CEP-1 pos	42% (92/221)	54% (93/173)	1.6 [1.1-2.4]	**p=0.02**
Cit-Fibβ_36-52_ pos	47% (104/221)	58% (101/173)	1.6 [1.1-2.4]	**p=0.03**
Cit-Fibα_563-583_ pos	42% (93/221)	52% (90/173)	1.5 [1-2.2]	NS (p=0.053)
Cit-Fibα_580-600_ pos	25% (55/221)	28% (49/173)	1.1 [0.76-1.9]	NS (p=0.49)
Cit-Fibα_621-635_ pos	30% (66/221)	39% (67/172)	1.5 [0.98-2.3]	NS (p=0.07)
Peptides with other motifs:				
Cit-Vim_60-75_ pos	45% (99/221)	51% (89/173)	1.3 [0.87-1.95]	NS (p=0.22)
Cit-Vim_2-17_ pos	35% (77/221)	38% (65/173)	1.1 [0.74-1.7]	NS (p=0.60)
Cit-Fibα_36-50_ pos	12% (27/221)	21% (36/172)	1.9 [1.1-3.3]	**p=0.03**
Cit-Fibβ_60-74_ pos	58% (127/221)	69% (118/172)	1.6 [1.1-2.4]	**p=0.03**
Number of ACPA fine-specificities (of 10)	3.7 ± 3.3 [3]	4.6 ± 3.2 [5]		**p=0.01**
***AMPA IgG specificities***				
ΔAc-His2B_6-22_ pos	15% (35/227)	19% (33/176)	1.2 [0.70-2.1]	NS (0.59)
Carb-CEP1 pos	22% (50/221)	35% (60/173)	1.8 [1.1-2.8]	**p=0.009**
mod-Vim IgG Orn(Ac) pos	53% (120/226)	60% (105/175)	1.3 [0.89-2.0]	NS (p=0.19)
mod-Vim IgG Carb pos	46% (105/226)	52% (91/175)	1.2 [0.84-1.9]	NS (p=0.31)
mod-Vim IgG Lys(Ac) pos	32% (72/226)	34% (60/175)	1.1 [0.73-1.7]	NS (p=0.67)
Carb-Fib pos	41% (92/226)	49% (86/175)	1.4 [0.95-2.0]	NS (p=0.1)
Carb-FCS pos	32% (72/226)	47% (83/175)	1.9 [1.3-2.9]	**p=0.002**
***RF***				
IgG pos	58% (131/227)	63% (111/176)	1.3 [0.84-1.9]	NS (0.31)
IgA pos	40% (90/227)	42% (74/176)	1.1 [0.74-1.65]	NS (0.68)
IgM pos	70% (158/227)	73% (128/176)	1.2 [0.75-1.8]	NS (0.51)

#Cutoff 56.44 (85^th^ percentile of 316 controls). *p-value from Fisher's exact test or Mann-Whitney analysis.

OR, Odds ratio; CI, 95% confidence interval; NS, Not statistically significant. Significant p-values are highlighted in bold font.

### Associations Between Different AMPA Reactivities Illustrate the Overlap With Citrulline Reactivity

**Figure 5:**
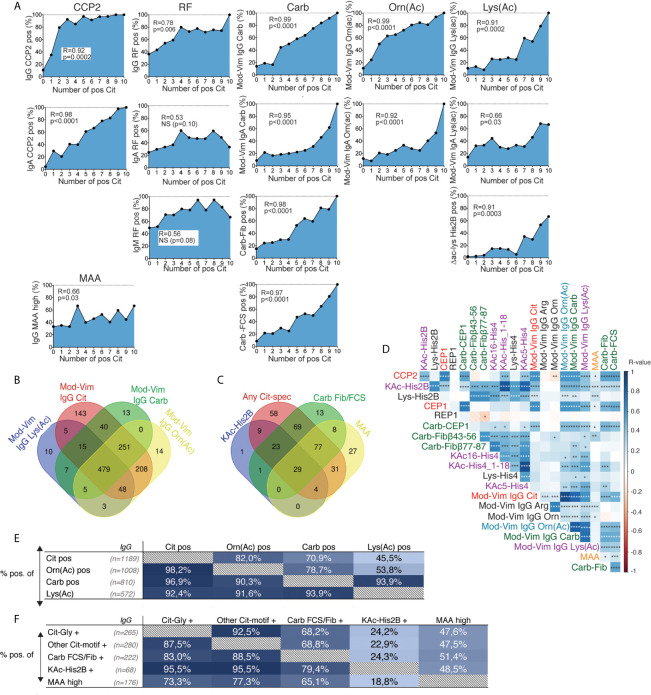
Correlation between different IgG AMPA reactivities in RA. **(A)** Correlation of the frequency of autoantibody positivity with the number of IgG ACPA fine-specificities by autoantigen microarray screening (based on 10 Cit-peptides) in 402 RA patients. P-values and R-values are presented from Spearman correlation. **(B)** Venn diagram for positivity in the mod-Vim assays among 1984 RA patients **(C)** Venn diagram for positivity among other IgG AMPA reactivities in 393 RA patients. **(D)** Kendall correlation matrix for continuous measures of serum antibody levels in different AMPA tests and control assays (in 80-402 RA patients). *p < 0.05, **p < 0.01, ***p < 0.001, ****p < 0.0001. Red color represents a negative R-value and blue color represents a positive R-value. **(E)** Distribution of mod-Vim IgG AMPA assay positivity among 1984 RA patients. **(F)** Distribution of AMPA assay positivity among 402 RA patients comparing: Cit-peptide reactivity (any positivity among six peptides with the Cit-Gly motif, any positivity among four peptides with other Cit-motifs), any anti-Carb-fibrinogen or anti-Carb-FCS positivity, positivity for IgG anti-acetylated histone 2B, or high levels of IgG anti-MAA. The frequency (%) of positive patients for the assay, shown in columns (x-axis), are presented for the patient groups, shown in rows (y-axis).

When collectively analyzing the different IgG AMPA reactivities we observed that anti-Carb and anti-KAc reactivity by the different assays strongly correlated with the number of IgG ACPA fine-specificities by array analysis (**Figure 5A**). These associations were similar to what was also seen for nearly all (9/10) individual IgG ACPA fine-specificities, but different from what was seen for RF (**Figure 5A** and **Supplemental Figure 8**). IgG anti-MAA demonstrated a lower correlation with the number of ACPA fine-specificities.

Anti-Carb and anti-KAc reactivities primarily co-existed with citrulline reactivity (**Figure 5** and **Supplemental Figures 9, 10**). Thus, almost all IgG Orn(Ac), Carb, and Lys(Ac) IgG positive patients were IgG anti-Cit positive. We also observed an association with CCP2 IgG levels, where patients with very high CCP2 levels (>1000 AU) had a higher frequency of AMPA positivity by the mod-Vim assays (**Supplemental Figure 11**). Furthermore, most KAc positivity was detected within the Carb-positive subset.

We have previously shown that many monoclonal ACPA+ antibodies generated from individual B-cell clones have preferential binding to Cit-peptides that display a citrulline-glycine (Cit-Gly) motif, which is also a fairly dominating reactivity in CCP2 affinity chromatography purified polyclonal IgG (26). Many of the Cit-peptides that were included in the array assay carry this Cit-Gly motif (**Supplemental Table 1**). We therefore made sub-analyses, dichotomizing the patients by reactivity to Cit-Gly peptides (six peptides) and by reactivity to other Cit-peptides (four peptides). However, we could not find any evidence that indicated that anti-Carb or anti-KAc reactivity more commonly associated with one or the other anti-peptide reactivity group but it should be noted that there was a large overlap between Cit-reactivities. Hence, a majority of ACPA+ patients were positive for several ACPA fine-specificities from both peptide groups. Furthermore, for CCP2 negative ACPA fine-specificity positive RA patients, there were no difference in positivity between the two peptide subsets (**Figure 5E** and **Supplemental Figure 9**). High anti-MAA reactivity showed overlapping positivity with the other autoantibody tests but was still the test that was most independent. Similarly, while the different IgG anti-Carb, -Cit, and -KAc AMPA serum levels all correlate strongly, IgG anti-MAA levels correlated to a lesser extent with the other AMPA reactivities in Kendall analysis (**Figure 5D**).

Notably, adding all antibody tests substantially reduced the true seronegative patient subset. The correlation between the different AMPA reactivities was similar in CCP2- as in CCP2+ patients, and positivity for several tests co-existed also in seronegative RA (**Supplemental Figure 10**). Among the 402 RA patients included in the KAc-His2B and anti-MAA screening, 70 individuals (17.4%) were seronegative for both CCP2 IgG and RF IgM, the tests used in the clinic, but of these only 17 (i.e. 4.2% of the total RA study population) were negative in all available autoantibody tests.

### IgA AMPA Reactivity Is Detected in a Limited Subset of Patients With IgG Autoantibodies

**Figure 6:**
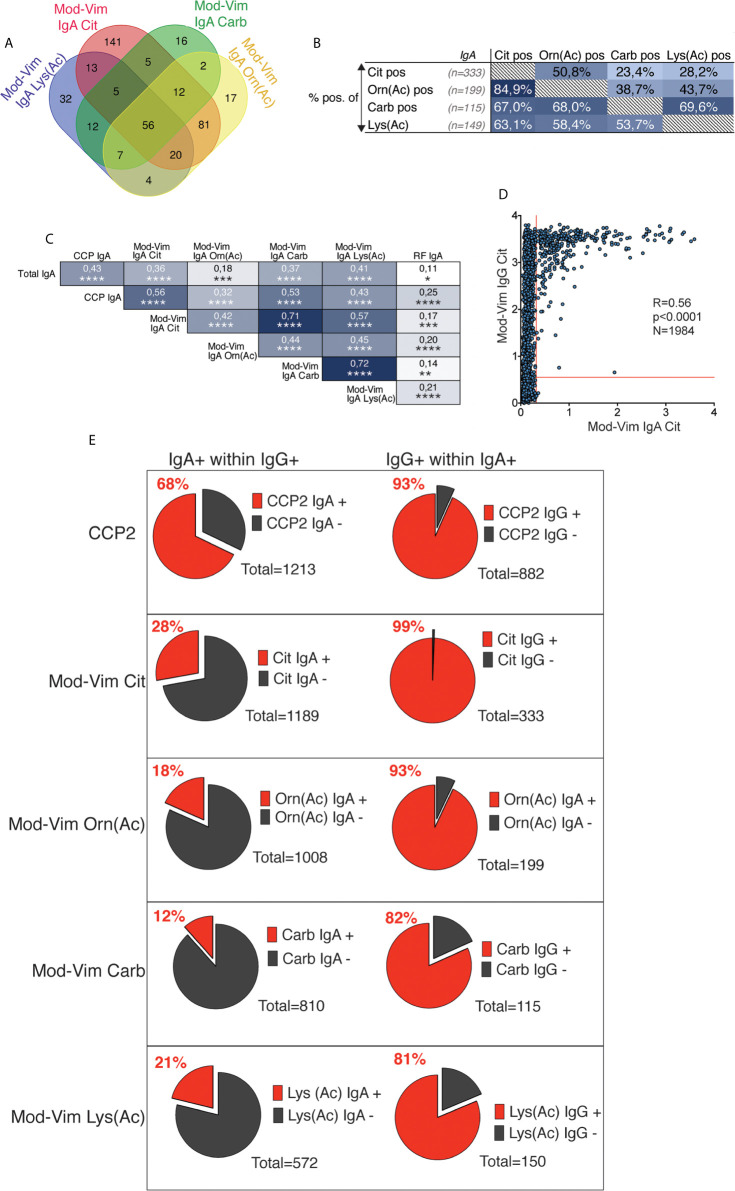
Correlation between different IgA AMPA reactivities in RA. **(A)** Venn diagram for AMPA IgA positivity by mod-Vim assays among 1984 RA patients. **(B)** Distribution of mod-Vim IgA AMPA assay positivity among 1984 RA patients. The frequency (%) of positive patients for the assay, shown in columns (x-axis), are presented for the patient groups, shown in rows (y-axis). **(C)** Spearman correlation of IgG and IgA AMPA reactivity in 402 RA patients. **(D)** Spearman correlation of mod-Vim citrulline IgG compared to IgA reactivity in 1983 RA patients. **(E)** Frequencies of IgA+ within IgG+ patients (left panels) and frequencies of IgG+ within IgA+ patients (right panels) for the same assays.

In the current study, the mod-Vim assays and CCP2 IgA enabled a direct comparison between IgG and IgA AMPA reactivities. Similar to the IgG reactivity, there was a considerable overlap between the different IgA specificities (**Figure 6**). However, while >90% of mod-Vim IgG anti-Carb, anti-Orn(Ac) and anti-Lys(Ac) were mod-Vim anti-Cit IgG positive, only 63-85% of IgA anti-Carb/Orn(Ac) or Lys(Ac) positive patients were mod-Vim anti-Cit IgA positive. We instead observed a stronger correlation between IgG and IgA to the same modified peptide. Firstly, the IgA Cit-reactivity was primarily detected in individuals with high levels of IgG Cit-reactivity (**Figure 6D**) and for each IgA AMPA test a majority (81-99%) were positive for the equivalent IgG test (**Figure 6E**). While IgA CCP2 positivity was common (68% of IgG CCP2+), the IgA mod-Vim anti-Cit, anti-Orn(Ac), anti-Carb, anti-Lys(Ac) positivity was seen in a smaller subset (12-28% of the corresponding mod-Vim IgG+). We also demonstrated that IgA AMPA positivity is associated with high CCP2 IgG levels (**Supplemental Figure 11**) and while this was also seen for AMPA IgG, it was more striking for AMPA IgA (**Supplemental Figures 11** and **12**). Some patients would have multiple IgA reactivities but there was no evidence that certain IgG AMPA positivity would be associated with higher probability of multiple IgA peptide reactivities (**Supplemental Figure 12**).

While there was a strong association with HLA SE and positivity in the different mod-Vim IgG AMPA assays, but only IgA anti-Cit and anti-Orn(Ac) displayed a significant association among the IgA test. When including only CCP2 positive patients in the analysis, no significant association was seen between HLA SE positivity and IgA AMPA (**Supplemental Table 4**).

Interestingly, similarly to IgA anti-CCP2 (44), mod-Vim IgA anti-Cit, Orn(Ac), and Carb positive frequencies were significantly increased in CCP2+ IgG RA patients that had a history of smoking (**Supplemental Table 3**). We did not observe an equivalent association with smoking for patients positive in the mod-Vim AMPA IgG assays. The history of smoking was significantly higher in IgG IgA AMPA double positive individuals compared to patients with only IgG positivity (**Table 4**). Although these patients in addition had higher CCP2 IgG (**Supplemental Table 8**), the association remained significant also when adjusting for CCP2 IgG levels. The association was strongest for IgA anti-citrulline, lower for IgA anti-Carb and Orn(Ac) and not significant for IgA anti-Lys(Ac).

**Table 4:** Frequency of smoking in AMPA IgG+ patients with and without IgA autoantibodies.

	IgG+ IgA-	IgG+ IgA+	*OR [CI]*	p-value*	Adjusted p-value**
CCP2	49% (189/386)	66% (569/859)	2.0 [1.6-2.6]	**<0.0001**	**<0.0001**
mod-Vim Cit	57% (483/855)	71% (237/331)	1.9 [1.5-2.6]	**<0.0001**	**<0.0001**
mod-Vim Orn(Ac)	60% (489/821)	71% (131/185)	1.6 [1.1-2.3]	**0.004**	**0.01**
mod-Vim Carb	60% (430/713)	72% (68/94)	1.7 [1.1-2.8]	**0.02**	NS (0.055)
mod-Vim Lys (Ac)	59% (263/447)	64% (78/121)	1.27 [0.84-1.93]	NS (p=0.30)	

*p-value from Fisher’s exact test.

**p-value from logistic regression analysis adjusting for CCP2 IgG levels.

OR, Odds ratio; CI, 95% confidence interval; NS, Not statistically significant. Significant p-values are highlighted in bold font.

### Cluster Analysis of AMPA Reactivities Reveals Different Patient Subsets

**Figure 7:**
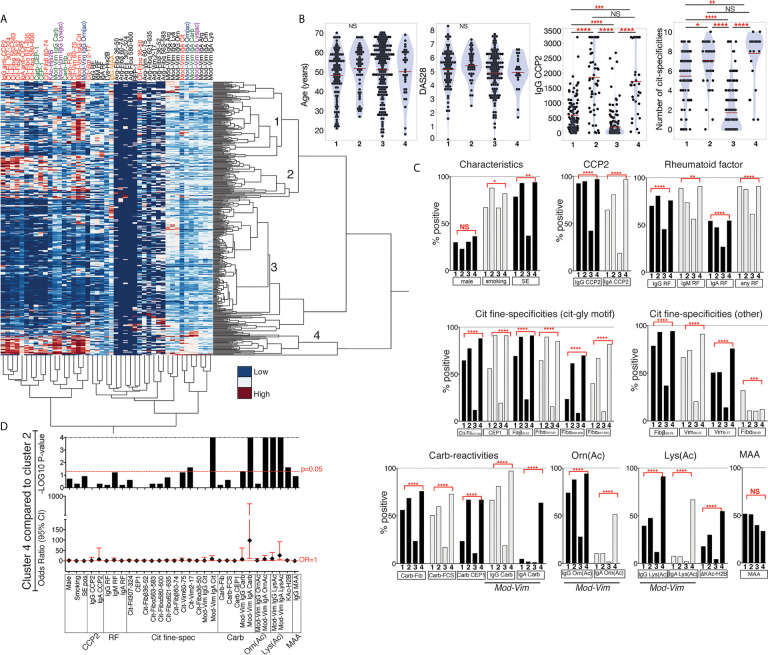
Cluster analysis reveals different subsets of RA patients with AMPA reactivity. **(A)** Hierarchical clustering of autoantibody reactivities in 384 RA patients using Ward’s method and normalized values. Four clusters of patients were further analyzed, with cluster 3 (n = 187) being primarily ACPA negative/low, and cluster 1 (n = 107), cluster 2 (n = 57) and cluster 4 (n = 33) with higher autoantibody positivity. **(B)** Differences in age, DAS28 disease activity, IgG anti-CCP2 and number or ACPA fine-specificities (of 10 tested) in the different clusters. P-values are indicated from Kruskal-Wallis analysis with Dunn’s correction for multiple comparisons. **(C)** Comparison of frequency of antibody positivity in the different patient clusters. P-values are indicated from logistic regression analysis, showing if the three ACPA+ clusters (1, 2 and 4) are significantly different when adjusting for age, HLA shared epitope, sex, smoking, and DAS28. **(D)** Comparison of autoantibody positivity and characteristics between cluster 2 and 4 using Fisher’s exact test.

We used hierarchical clustering to compare all antibody reactivities in 402 RA patients (**Figure 7**). This analysis resulted in four different patient clusters, with cluster 3 capturing mostly antibody negative/low individuals, and cluster 1, 2 and 4 including CCP2 positive patients. While the CCP2 levels were higher in clusters 2 and 4, there was no significant difference in age, sex, or baseline DAS28 between the clusters. ANOVA analysis did also not show any significant DAS28 differences between clusters at six months follow up (data not shown). A history of smoking was more frequent in clusters 2 and 4 and the frequency of HLA shared epitope positivity was, as expected, lower in the antibody low/negative cluster 3. In regression analysis most antibody tests were significantly different between the three CCP2+ patient clusters (1, 2 and 4) also when adjusting for potential co-factors (age, sex, *HLA* SE, smoking, and DAS28), with the exception of IgG anti-MAA (**Figure 7C**). Patients in cluster 1 displayed fewer ACPA fine-specificities while clusters 2 and 4 had similar numbers. Interestingly, when looking more closely at the differences between cluster 2 and 4, it was evident that patients in cluster 4 had significantly higher AMPA positivity and especially high frequency of lysine-acetylation reactivity and IgA Carb, Orn(Ac) and Lys(Ac) positivity (**Figure 7D**).

In conclusion, there is substantial co-occurrence of different AMPA reactivities in RA. We can identify subsets of RA patients with different AMPA profiles. Specifically, one RA patient subset had particularly high Cit-reactivity and anti-Carb/KAc IgG and IgA AMPA multireactivity.

### AMPA Reactivity in Individual B-Cell Clones Support the Serology Patterns

**Figure 8:**
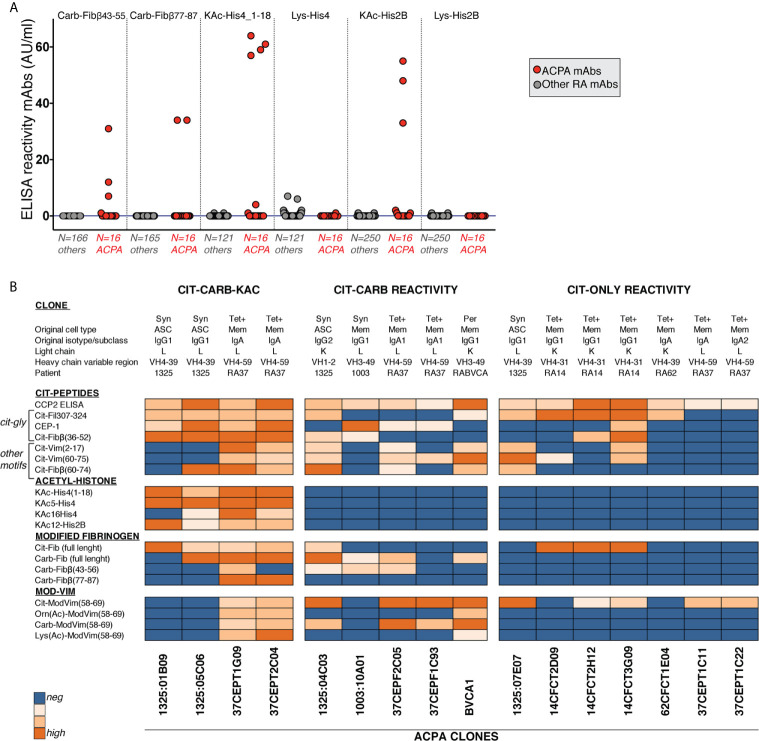
Screening of RA B cell-derived monoclonal antibodies show acetyl-lysine and carbamylation reactivity only in CCP2+ clones. **(A)** Recombinant monoclonal antibodies derived from single B cells from RA patients were screened for reactivity to carbamylated and citrullinated antigens by ELISA at 5µg/ml IgG. 250 clones had no known citrulline reactivity (others) and 16 clones were positive for binding in CCP2 ELISA (ACPA mAbs). Clones showing unspecific binding in any poly-reactivity assay were excluded before screening. **(B)** Reactivity pattern of 16 monoclonal anti-CCP2 positive ACPA clones to different modified antigens. Data is derived from the ACPA fine-specificity microarray (Cit-peptides) or ELISA (Carb/KAc and mod-Vim assays).

**Figure 9:**
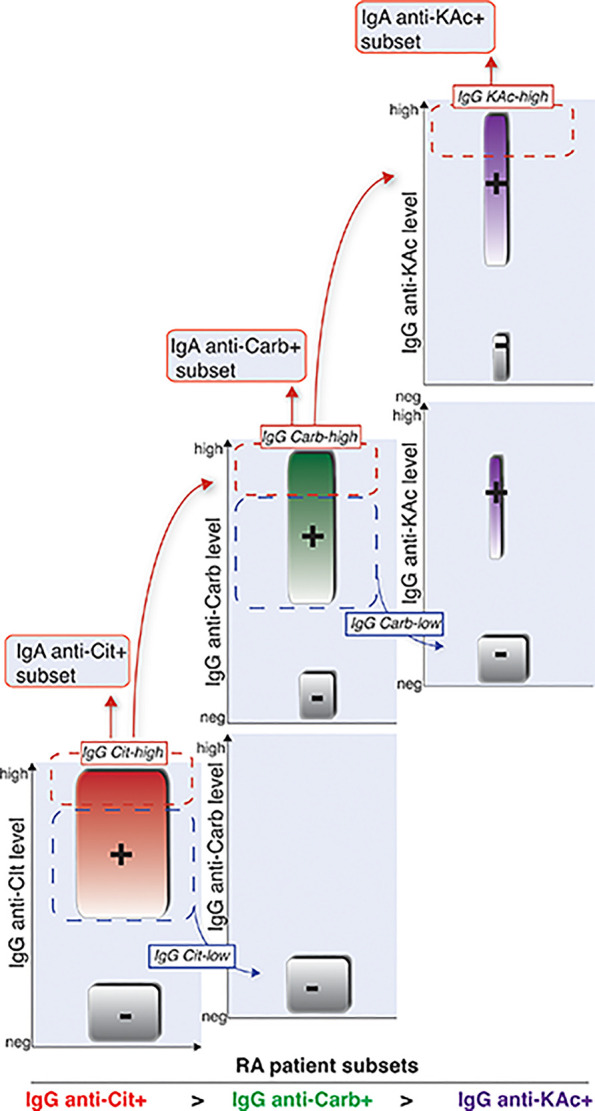
Schematic illustration of how different RA AMPA+ patient subsets are related. Graphical visualization of how we interpret that the different AMPA subsets are related in RA patients. Cit-reactivity represents the largest subset followed by Carb-reactivity. KAc is observed in a smaller subset of RA patients. The IgG-Carb reactivity is more frequent in RA patients with high detectable levels of Cit-reactivity, and KAc is more frequent in patients with Cit and Carb positivity. Similarly, IgA reactivity is observed in smaller subsets of RA patients with high IgG levels of the same AMPA reactivity.

We have in previous studies identified RA patient derived CCP2+ ACPA from a large number of generated human monoclonal antibodies (>250) from single B-cells from different tissues from seropositive RA patients (26, 27, 32, 41, 48, 49). One of the most striking features of these ACPA clones is the extensive multireactivity to modified-peptides and proteins (26, 27). In **Figure 8**, we extend the investigation of multireactivity to Cit- Carb- and KAc-peptides of 16 different human ACPA mAbs and demonstrate that the clones can be divided into three different groups: i) Cit-only reactive, ii) Cit-Carb multireactive and iii) Cit-Carb-KAc multireactive, based on screening using the same peptides as for the serology investigation. Additionally, we investigated the non-ACPA clones without CCP or Cit-peptide reactivity. Notably, no clone could be identified with only Carb or KAc binding i.e. without CCP2 and citrulline binding (**Figure 8A**). This monoclonal data set supports the serology data, that autoantibodies to carbamylated and acetylated epitopes should be considered AMPA fine-specificities within the citrulline-reactivity family and not independent autoreactivities. Our interpretation of the relation between Cit, Carb and KAc IgG and IgA reactivity are summarized in **Figure 9**.

## Discussion

Rheumatoid arthritis is associated with autoreactivity to post-translational modifications of proteins. While citrulline-reactivity has been subject to most RA studies, a number of other interesting autoreactivities have emerged. In this study we dissect the relation between anti-citrulline, anti-carbamylation, anti-acetylation and anti-malondialdehyde adducts reactivities. Among these, the anti-MDA/MAA autoreactivity stands out as not being RA-specific and not showing as strong association with ACPA fine-specificities as the other AMPA. Autoreactivity to carbamylated and acetylated epitopes on the other hand, is more commonly found within RA patients with citrulline-reactivity. The multireactivity properties found in ACPA mAbs, together with these serology patterns, suggest that anti-Carb and anti-KAc antibodies should be considered another flavor of ACPA/AMPA fine-specificities. They are associated with high CCP2 levels and high number of positive ACPA fine-specificities, but cluster analyses show that there are both RA subsets with high CCP2 and AMPA multireactivity and RA subsets with high CCP2 without AMPA. Reactivity to acetylated-lysine peptides is observed in a substantially smaller patient subset and mostly overlapping not only with citrulline reactivity but also with anti-Carb. Moreover, we find that IgA citrulline, acetylation and carbamylation reactivities are primarily found in a subset of RA patients with high IgG anti-citrulline reactivity by CCP2 and/or the mod-Vim Cit assays. While a majority of the IgA+ patients were positive for IgG to the same modification, IgA was only found in smaller subsets of the IgG PTM+ patients using the modified Vim-peptide assays.

IgA is an important isotype in mucosal responses, yet IgA is also abundant in the circulation. The IgA1 subclass is more dominating in the circulation (9:1 ratio) but RA patient have been reported to have slightly higher IgA2 ratio, which was also suggested to be more pro-inflammatory (53). Importantly, in the Ig locus, the α1 and α2 constant region elements are located downstream of the µ, γ3 and γ1 constant regions. Consequently, IgM, IgG3 and IgG1 can class-switch to IgA but not the opposite. ACPA IgG has previously been shown to be primarily IgG1 and to a lesser extent IgG4 (54). Although class-switching is regulated by several layers of mechanisms, including the local milieu in tissue sites, more germinal center rounds may also result in higher probability that a B-cell clone may class-switch all the way to IgA. Certain paths are also more frequent (e.g. more IgG1 to IgA1, compared to IgG3 to IgA1) (55). This is also reflected by on average higher somatic hypermutation (SHM) level in IgA+ than in IgG1+ circulating cells (46). Hence, we speculate that high IgG anti-CCP2 levels indicates a high ongoing chronic ACPA response that may promote IgG to IgA class-switching. IgG4, being downstream of IgG1, may be a result of similar mechanism(s). Class-switching to IgG4 and IgA, which are generally not FcR-activating, could be a normal regulatory mechanism as a result of chronic immune responses. Interesting, we have previously seen that IgA+ B-cell frequencies among class-switched cells are lower in RA compared to healthy controls, while serum IgA levels are higher (46). It has also been reported that IgA+ plasmablasts are elevated in antibody positive RA-risk individuals (56). However, serum IgA anti-CCP levels are increasing closer to disease onset compared to IgG anti-CCP, which could suggest that serum ACPA IgA is related to the chronic adaptive immune response, rather than to the initiation (57).

Since smoking is a strong risk factor for antibody positive RA and the mucosal interface in the lungs has been pinpointed as a likely candidate site for initiation of autoreactivity in RA (58), we also investigated IgA and IgG AMPA in relation to smoking. Previous studies have shown that smoking was overrepresented in IgA CCP2+ RA patients (44). Here we show that IgA anti-Cit-Vim, anti-Carb-Vim, and Orn(Ac)-Vim was significantly higher in smokers compared to non-smokers and IgG/IgA double positive patients were more likely to have a history of smoking. Although also high CCP2 IgG levels were associated with both smoking and IgA AMPA, the association between smoking and IgA AMPA was not dependent on IgG anti-CCP2 levels. We also observed that IgG anti-KAc-His2B IgG anti-Carb-FCS had significant correlation with smoking status in CCP2+ RA, however in this case only anti-Carb stayed significant when adjusting for IgG anti-CCP2. Hence, these results implicate that smokers have both higher total ACPA levels, and are more likely to have IgG Carb and KAc specificities as well as IgA ACPA/AMPA reactivity compared to non-smokers. However, there was no indication that IgA reactivity to any non-citrulline modification would have stronger association with smoking than IgA anti-Cit. Notably, we found that IgG RF was significantly associated with ever smoking in CCP2+ RA, and remained significant after adjustment for high CCP2 IgG levels. Interestingly, it has been reported that while there is an interaction of ACPA positivity, HLA-DRB1 SE, and smoking as risk factors for RA, the association of smoking with RA disease in RF+ CCP2- individuals is largely independent of HLA-DRB1 SE, suggesting different modes of induction (59, 60). Smoking may hence, besides being a possible trigger of disease, provide a continued immune stimulation that drives RF as well as IgG and IgA AMPA autoantibody production.

While most IgG AMPA reactivities were present in higher frequency in RA patients carrying HLA-DRB1 SE alleles, only a few remained significantly higher when analyzing only CCP2+ RA (i.e. IgG anti-Cit, anti-Orn(Ac) and anti-Carb). The association was weaker for IgA and among the non-citrulline reactivities and only IgA anti-Orn(Ac) was significantly associated with HLA-DRB1 SE in the whole RA cohort and no association was detected in CCP2+ RA. These results may implicate that the shared epitope genetic predisposition is primarily important for IgG anti-citrulline reactivity. Interestingly, while IgG anti-KAc-His2B was not associated with HLA-DRB1 SE in the RA cohort with or without subdividing based on CCP2 we could observe a negative association with HLA-DRB1*03 among CCP2+ patients that was to some extent dependent on IgG anti-CCP2 levels. This notion is supported by previous studies showing that there is a negative association between HLA-DRB1*03 and CCP2+ RA (61, 62).

Several investigations of autoantibodies to carbamylated and acetylated peptides and proteins in RA have been performed (20, 21, 23, 40, 63). We used different strategies for investigating reactivity to acetylated epitopes. Firstly, we focused on naturally occurring modified sites, for example in acetylated histone 2B K12 and histone 4 K5 and K16 that can occur during neutrophil extracellular trap formation (NETosis) (64). These sites have previously also been reported to be autoantibody targets in SLE (65), but in our studies the SLE reactivity was primarily to the unmodified histone epitopes while RA had reactivity to acetylated histones. We have recently revealed an association of monoclonal AMPA binding to these peptides and nuclear and NET reactivity (32). However, reactivity to these acetylated histone peptides are not unique, and RA reactivity to another KAc-His site has previously been published (23). In our second approach, the mod-Vim system had the advantage of enabling direct comparison between reactivity to different PTMs with a consistent peptide backbone (23). Since this require the original arginine to be replaced by a modified lysine, the generated epitope will be artificial and not representing the original antigen (i.e. vimentin) or a known autoantigen. Yet, the reactivity was low in control populations and the assays seem to capture an RA-specific AMPA autoimmune profile.

In our recent reports, we demonstrate that monoclonal human ACPA have a multireactivity to hundreds or thousands of different modified peptides and proteins (26, 27). The Cit-peptide binding was primarily dependent on only a few amino acid residues adjacent to the modified site. For some clones this motif-dependent multireactivity extended to Carb and KAc peptides. Notably, many clones recognize a Cit-Gly motif with varying preference in the -1 position, and this was also the most dominating motif in polyclonal anti-CCP2 preparations (26). The Cit-Gly motif is recurrent in several commonly used peptides from fibrinogen, filaggrin, and α-enolase. However, there are also a series of other motifs that can be recognized, and a preference for serine adjacent residues have been found to be frequent in serum screening using a similar peptide array approach (66). The mod-Vim assays, being based on a vimentin peptide, are displaying a Thr-X-Ser motif and may capture a different subset of antibodies than for example the His2B peptide with a Lys-KAc-Gly motif. Nevertheless, it should be noted that when dichotomizing the serum, citrullinated peptide reactivity in Cit-Gly *vs.* peptides with other motifs, a majority of patients displayed ACPA fine-specificity reactivities in both groups and there was no particular association between Cit-Gly or other motifs with Carb/KAc positivity. Hence, Carb or KAc autoreactivity could so far not be related to any specific Cit-motif group in our RA cohort.

Interestingly, although the recognition is not yet as well described as for the ACPA, studies of monoclonal anti-MDA/MAA antibodies from RA patients show that these autoantibodies also display a multireactivity to different modified proteins. The reactivity was equally strong to modified bovine serum albumin compared to human serum albumin and a range of other human proteins with some variation depending on mAb clone, suggesting that the RA antibodies are primarily targeting the modified amino acids (19). Several of the screening assays that we included in this study were similarly based on modified full-length proteins that can be expected to have a high degree of modification (i.e. carbamylated fibrinogen, carbamylated FCS or MAA modified BSA). While the proteins can be considered surrogate antigens and the assays are designed to detect reactivity to the modification, we acknowledge that there is also a possibility that the modification process introduce structural changes and expose cryptic neo-epitopes that can be targeted by antibodies.

Citrullination is an enzymatic reaction mediated by PADs that convert arginine to citrulline, while carbamylation is a non-enzymatic process catalyzed by cyanate and modifying lysine. Nevertheless, structurally, these two residues have similar biochemical properties and irreversibly modify proteins. Lysine acetylation is also generating an alteration from positive to neutral charge but is more strictly regulated by acetylases and deacetylases. Interestingly, all three modifications are increased during inflammation and during NETosis (64, 67, 68) and could hypothetically trigger independent autoantibody responses. Acetylation of ornithine (that was included in one of the assays) may on the other hand not be present in proteins *in vivo* and the assay also seems to capture very similar pattern to the citrulline assay. Cross-reactivity and overlap of autoantibodies to citrulline, homocitrulline and acetyl-lysine have been suggested in multiple studies (26, 27, 31, 32, 40, 69, 70) and immunization experiments in rabbits and mice have shown that one modification can give rise to antibodies to the other modification (69, 71). However, we here demonstrate that not all B-cell clones have these properties and not all ACPA+ RA patients display detectable autoreactivity to carbamylated and acetylated epitopes. In particular acetylation reactivity was primarily elevated in a smaller subset of patient, reflecting different polyclonal composition of these patients’ autoantibody repertoires. Furthermore, among RA monoclonal antibody clones, acetylation reactivity was only detected in conjunction with Cit and Carb reactivity. The clinical importance of high levels of IgG and IgA anti-acetylation autoreactivity remains to be elucidated. Previous studies have shown that the presence of anti-Carb can be associated with more severe disease (21) and reactivity to multiple modification correlate with higher risk of relapse when tapering DMARD treatment (23). However, IgG anti-CCP2-, IgM RF- RA patients with anti-Carb at baseline had lower disease activity by DAS28 than anti-Carb negative in 48-month follow up after disease onset and standard of care DMARD treatment (52).

Autoreactivity to MAA showed a different pattern of expression compared to the other investigated AMPA specificities. Malondialdehyde is released during oxidative stress through lipid peroxidation and can cause a range of irreversible amino acid modification that can be targeted by autoantibodies [reviewed in (72)]. IgM anti-MDA and anti-MAA that cross-react also with oxidized low-density lipoprotein (LDL) and apoptotic cells have been extensively described as part of the natural antibody repertoire present from birth (73–75), and have been suggested to have homeostatic functionality [reviewed in (76)]. Inflammation and oxidative stress lead to an increase in MDA-modified proteins that may trigger elevated expression of both IgM and IgG anti-MDA/MAA. This could explain the association with disease activity and inflammatory biomarkers (19) as well as the fact that while levels are elevated in RA, they are also increased in a number of other conditions and a smaller subset of the population controls (43, 77). Our anti-MAA data in the Risk-RA cohort, with no difference compared to population controls, supports that IgG anti-MAA is associated with the inflammatory disease. This was different from anti-acetylated histone antibodies that were increased in the RA-risk population and may follow a similar pattern as has been reported for ACPA fine-specificities and carbamylation reactivity that can arise years before RA onset (78–81). However, a recent report has shown that anti-MAA can be elevated before onset of RA disease but that the increase in levels occurs later in the pre-clinical phase than other autoantibodies (82). Our previous data demonstrate that certain anti-MDA/MAA clones but not others may contribute to RA pathogenesis by osteoclast activation (19). Notably, in our hands we have not been able to clearly separate MDA from MAA-reactivity since *in vitro* antigen modification using only MDA commonly results in formation also of MAA residues due to release of acetaldehyde from MDA decomposition and the MAA protocol may also generate MDA residues. In addition, MDA and MAA treatment can result in different levels of intra-molecular crosslinking that effect antibody targeting. Nevertheless, the malondialdehyde-acetaldehyde (MAA) residues have been suggested to be the most immunogenic MDA-adducts (83). Reactivity to different MDA or MAA modified antigens, bovine serum albumin, human serum albumin, or LDL seem to follow similar patters with increased levels in RA (19, 22, 84) and consequently the PTM seems to be more important than the protein context for serum reactivity. Yet, use of different antigens may make different screening efforts difficult to compare.

In summary, MDA/MAA autoreactivity, while associated with antibody positive disease, is not directly related to citrulline reactivity. Anti-Carb and anti-KAc reactivity could on the other hand be considered to belong to the same umbrella autoreactivity family as different ACPA fine-specificities and most likely reflect the presence of multi-reactive clones. RA patients clustered in different sub-groups depending on their AMPA reactivity profiles, especially based on if they had acetylation reactivity and IgA AMPA specificities. Several pathogenic synovial functionalities of ACPA mediated by specific modified antigen binding have been proposed and demonstrated with polyclonal preparations *in vitro*, including immune complex formation with citrullinated fibrinogen (85, 86) or citrullinated histones (11, 32) stimulating TNF release, and anti-citrullinated vimentin ACPA mediating osteoclastogenesis (4). Interestingly, CCP2-reactive monoclonal antibodies with different AMPA multireactivity profiles seem to target and affect different cell types (i.e osteoclasts *vs*. fibroblasts) (6, 7, 27). We have also shown that AMPA mAbs with acetylated-histone reactivity can bind NETs differently than AMPA with only citrulline reactivity (32). Furthermore, rheumatoid factor, while not having a direct effect by itself, can work as amplifier of different AMPA inflammatory pathways (87). Hence, we speculate that the composition of an RA patient’s autoantibody serology profile may reflect what molecular and cellular pathways that are more likely to drive the disease in that particular patient. It is evident that RA patients have a heterogeneous autoreactivity response and that different RA patient subsets exists. We speculate that different AMPA profiles may be related to the immune activity status, disease progression, disease activity and/or severity in different stages of disease, and learning more about these potential associations will be important for better understanding of the heterogeneity of RA. Longitudinal investigations before and after therapeutic interventions in association with treatment responses will be especially interesting. Better understanding of the functions of different AMPA may also enable us to identify and target distinct effector functions of AMPA.

## Data Availability Statement

The raw data supporting the conclusions of this article are available from the corresponding author upon request.

## Ethics Statement

The studies involving human participants were reviewed and approved by the regional ethics review board in Stockholm, Sweden. The patients/participants provided their informed consent to participate in this study.

## Author Contributions

All authors were involved in drafting the article and revising it critically. CG, LL, VM, JR, AC, KL, and LK designed the studies and interpreted the data. CG and LL developed methodology, performed peptide AMPA serological screenings and analyzed the complete data set. MH, LM-A, LI, and KL performed additional serology screenings. HB conducted the Mod-Vim analysis at Orgentec Diagnostika. JR performed statistical analysis and contributed with valuable discussions. AH, VJ, and AC provided clinical samples and data for the Risk-RA cohort. VJ, AH, JS, LP, PT, KL, VM, and NS generated human monoclonal antibodies from single B-cells. RS produced and validated monoclonal antibodies. LL performed monoclonal AMPA screenings and CG and VM analyzed and interpreted results. ES and IG provided SLE samples, clinical data, and discussions. GS and CC provided cit-fibrinogen peptides and ACPA insights. AK and AS performed IgA anti-CCP2 analysis. LA and LK are the principal investigators for the EIRA early RA cohort, with sample collection, environmental and genetic data. SS and LA provided SRQ and EIRA data and discussions. AC, SS, and AH provided RA clinical insight. CG wrote the first manuscript draft. All authors contributed to the article and approved the submitted version.

## Funding

This work was supported by the Swedish Research Council (2013–03624, 2017-01696), the Swedish Rheumatism Association (R-856551; R-931809, R-931647), Åke Wiberg’s foundation (M15-0087, M16-0060, M17-0166), apotekare Hedberg’s foundation, King Gustaf V’s 80-year foundation (FAI-2018-0493, FAI-2019-0592), and the EU/EFPIA Innovative Medicines Initiative (IMI) 2 Joint Undertaking projects BTCure 115142 and RTCure 777357.

## Conflict of Interest

HB is an employee at Orgentec Diagnostika and LM-A is an employee at Thermo Fisher Scientific. KL is co-inventor of patent: US12/524,465, describing the diagnostic use of the CEP-1 epitope.

The remaining authors declare that the research was conducted in the absence of any commercial or financial relationships that could be construed as a potential conflict of interest.
