# Supplementary material for: A Comprehensive Evaluation of the Relationship Between Different IgG and IgA Anti-Modified Protein Autoantibodies in Rheumatoid Arthritis
Source: Front Immunol. 2021 May 20;12:627986. doi: 10.3389/fimmu.2021.627986 (PMC8173192; doi:10.3389/fimmu.2021.627986)
Supplement: Supplementary file 1 [file DataSheet_1.pdf]

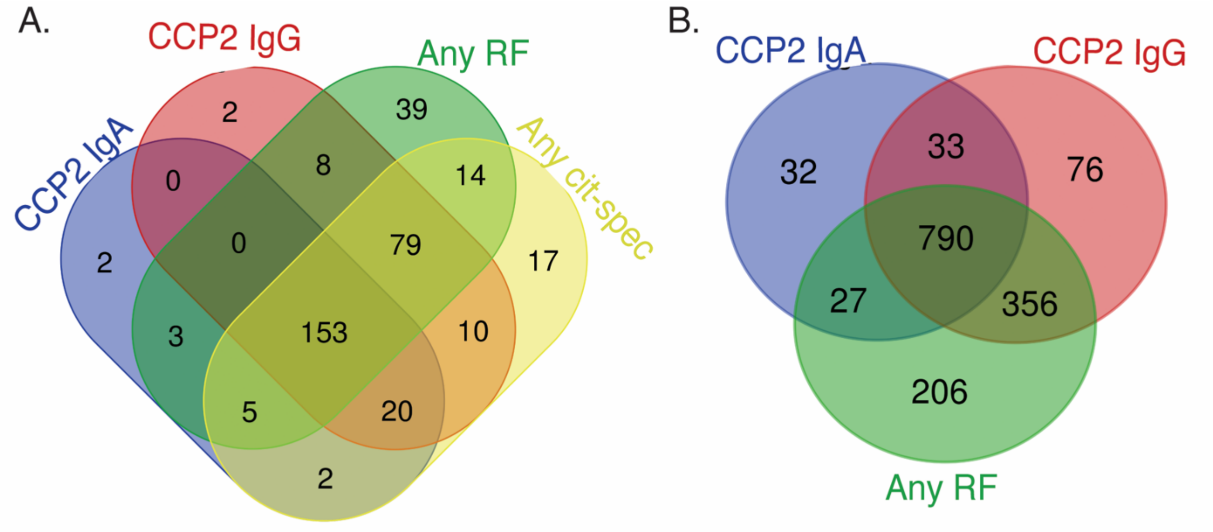

Supplemental Figure 1. Overlap of rheumatoid factor and citrulline reactivity in RA

A. Data is shown for autoantibody screening in of 386 patients comparing CCP2 IgG (CCPlus Euro Diagnostica), CCP2 IgA (as previously reported [1]), any rheumatoid factor (RF) isotype positivity (Phadia ELiA, IgM, IgG, IgA) and citrulline peptide reactivity by antigen microarray (Thermo Fisher Scientific, 10 Cit-peptides included, see Supplemental Table 1).
B. Data from 1942 patients with CCP2 IgG and IgA and RF IgG, IgA and IgM. Venn diagrams were generated using an online tool (http://bioinformatics.psb.ugent.be/webtools/Venn/).

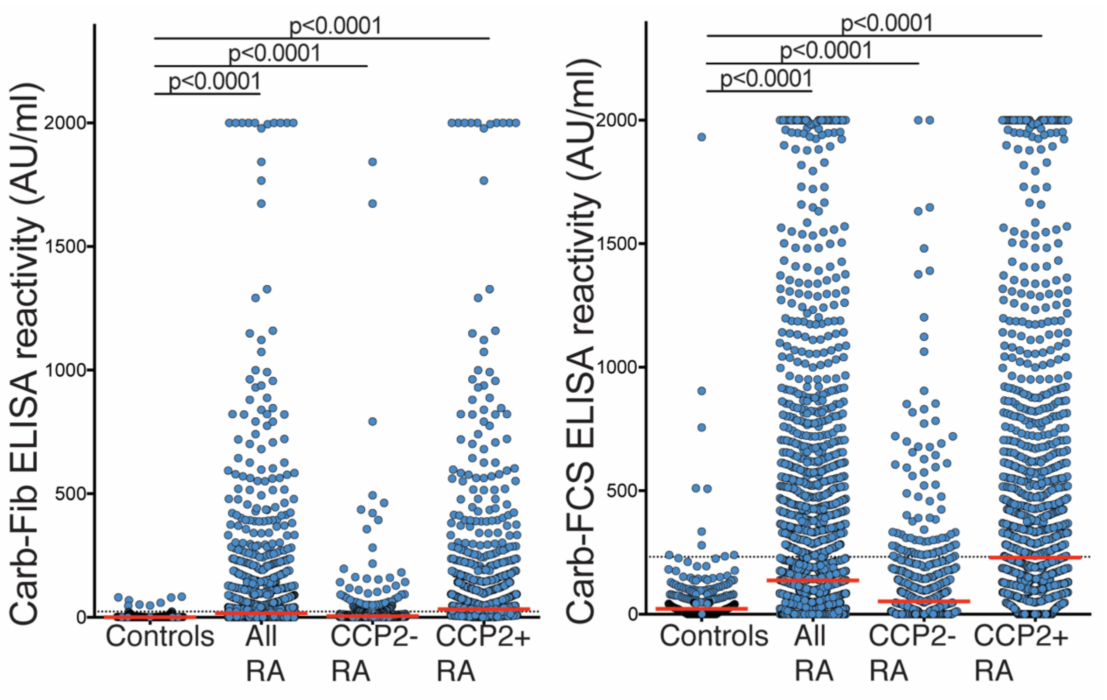

Supplemental Figure 2. Detection of IgG reactivity to carbamylated full-length protein

Results from previously published [2] carbamylated full-length fibrinogen and fetal calf serum (FCS) ELISA screening of 316 population controls and 1985 RA patients from the EIRA cohort, whereof 730 were ACPA negative by the CCP2 assay and 1255 were CCP2 positive. Red lines depict medians. P-values are presented from Kruskal-Wallis test with Dunn's correction for multiple comparisons.

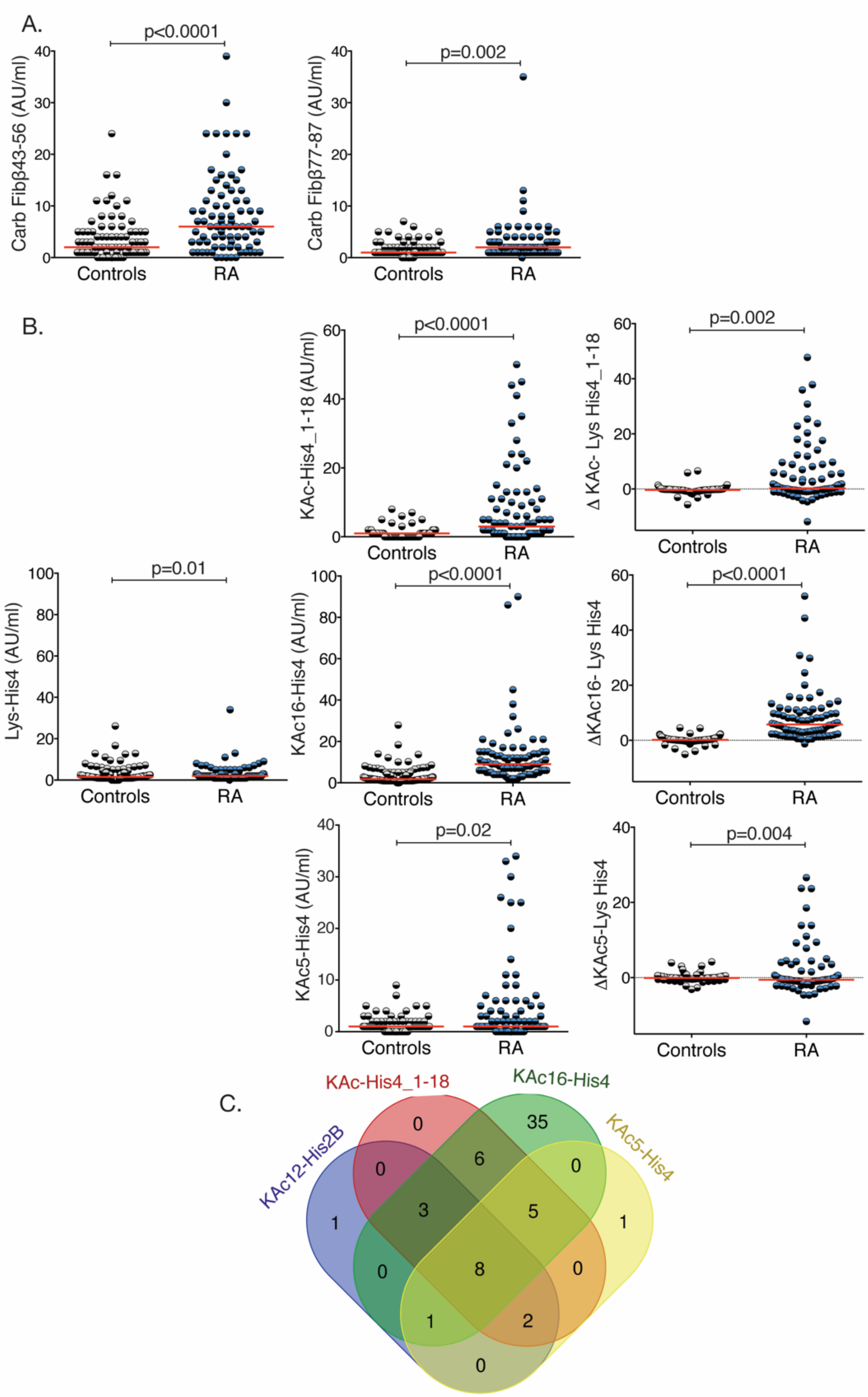

Supplemental Figure 3. Exploratory screening of other AMPA specificities

A. ELISA screening using two previously identified fibrinogen-derived carbamylated peptides [3] in 80 population controls and 80 RA patients. **B.** ELISA screening of reactivity to acetylated histone 4 in 200 population controls and 80 RA patients. Three different acetylated peptides were assessed, KAc-His4_1-18 with four modified sites, KAc5-His4 (with K5 modified) and KAc16 (with K16 modified) and compared to the native peptide, Lys-His4. P-values from Mann-Whitney analysis. **C.** Venn diagram showing the overlap in IgG positivity to different acetylated histone peptides in the 80 RA patients. Peptide sequences are available in Supplemental Table 1.

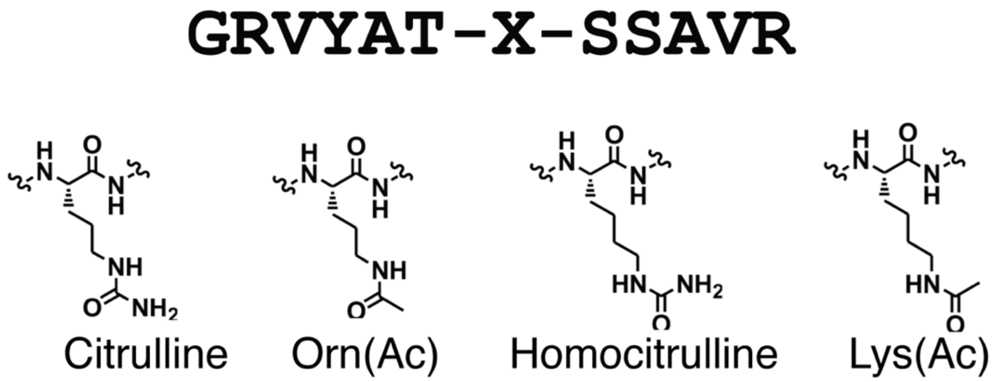

Supplemental Figure 4. Modifications in the modified-vimentin assay

The modified vimentin assay is based on a 12 amino acid vimentin sequence derived from the vimentin protein used in the MCV assay [4, 5]. The original arginine peptide is replaced by lysine, ornithine (Orn), homocitrulline (Carb), acetyl-ornithine (Orn(Ac)) or acetyl-lysine (Lys(Ac)) as previously reported [5]. Sequence of the peptide and structures of the different modification are shown in the figure with X marking the modified residue in the sequence.

A.

IgG autoAbs	IgG anti-MAA		IgG anti-KAc-His2B		IgG anti-Lys-His2B		Δac-lys His2B	
	R-value	*p-value*	*R-value*	*p-value*	*R-value*	*p-value*	*R-value*	*p-value*
RF	0,24	**0,01**	0,05	0,58	-0,08	0,35	0,22	**0,01**
Cardiolipin	0,34	**<0,0001**	0,26	**0,001**	0,24	**0,003**	-0,07	0,38
β2GP I	0,35	**<0,0001**	0,29	**0,0004**	0,27	**0,001**	-0,11	0,20
dsDNA	0,32	**<0,0001**	0,27	**0,001**	0,21	**0,01**	-0,14	0,08
Nucleosome	0,33	**<0,0001**	0,29	**0,0003**	0,29	**0,0004**	-0,17	**0,04**
Ribosome	0,11	0,20	-0,05	0,54	-0,06	0,47	-0,03	0,75
Sm	0,25	**0,002**	0,17	0,03	0,13	0,13	-0,01	0,95
RNP A	0,10	0,24	0,04	0,65	0,01	0,92	-0,02	0,80
SSA/Ro52	-0,09	0,27	-0,13	0,12	-0,13	0,12	0,03	0,74
SSA/Ro60	-0,11	0,19	-0,09	0,29	-0,14	0,10	0,10	0,24
SSB/La	-0,15	0,07	-0,09	0,30	-0,17	0,04	0,10	0,21

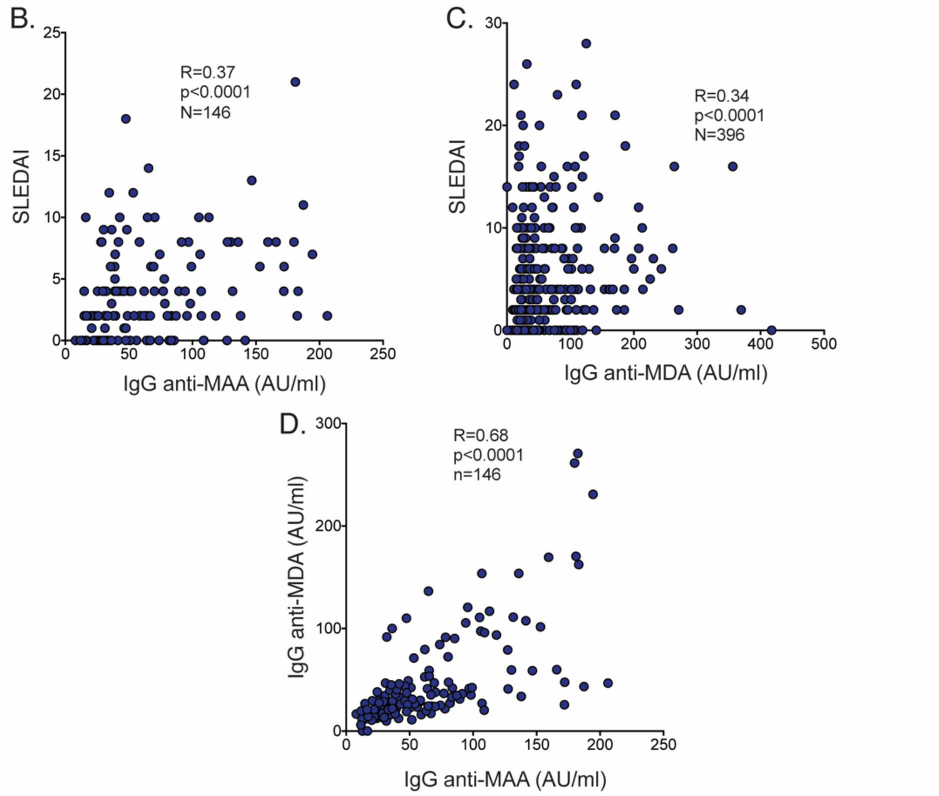

Supplemental Figure 5. Association of IgG anti-MAA and anti-His2B with other autoantibodies in patients with SLE

A. Spearman correlation between IgG anti-MAA, native histone 2B (aa 6-22) and acetylated histone 2B (K12) and different IgG autoantibody levels in 159 SLE patients from the Karolinska lupus cohort. IgG anti-MDA in SLE has previously been reported in Hardt et al[6]. Correlation between serum level of IgG anti-malondialdehyde acetaldehyde (MAA) modified BSA (**B**) or of IgG anti-malondialdehyde (MDA) BSA (**C**) by ELISA in SLE patients and disease activity by SLEDAI. **D**. Correlation between IgG anti-MDA and anti-MAA in SLE.

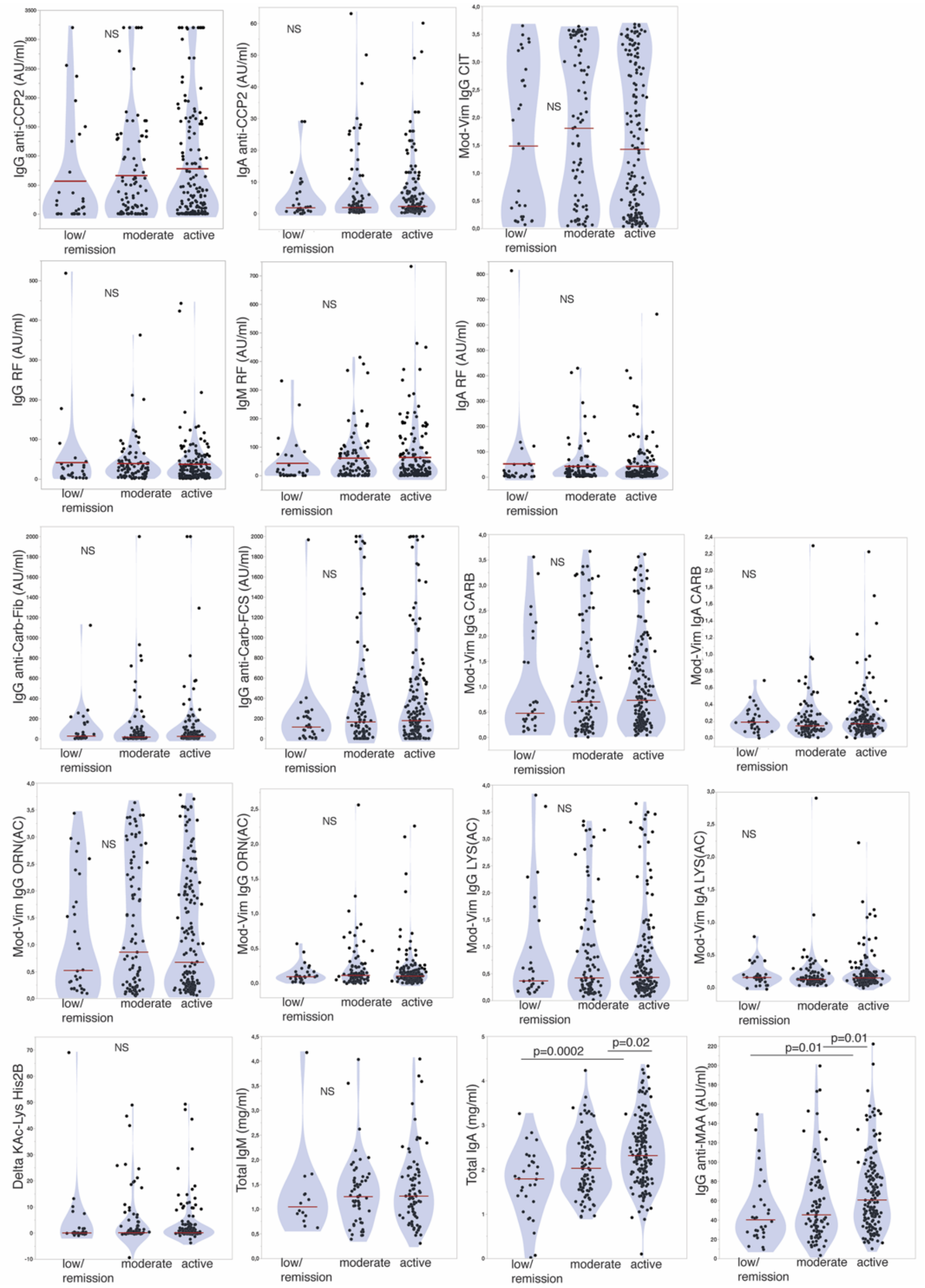

Supplemental Figure 6. Association of RF and different AMPA reactivities with disease activity by DAS28

AMPA reactivity in association with RA disease activity by DAS28 in 281 RA patients. 30 RA patients had low disease activity (DAS28<3.2), 93 had moderate disease activity (3.2 ≤ DAS28 ≤ 5.1) and 158 had high disease activity (DAS28>5.1). All RA patients had early disease without DMARD treatment. Red lines depict medians. P-values are presented from Kruskal-Wallis test with Dunn's correction for multiple comparisons. Only the IgG anti-MAA levels and total IgA levels were significantly different between the groups.

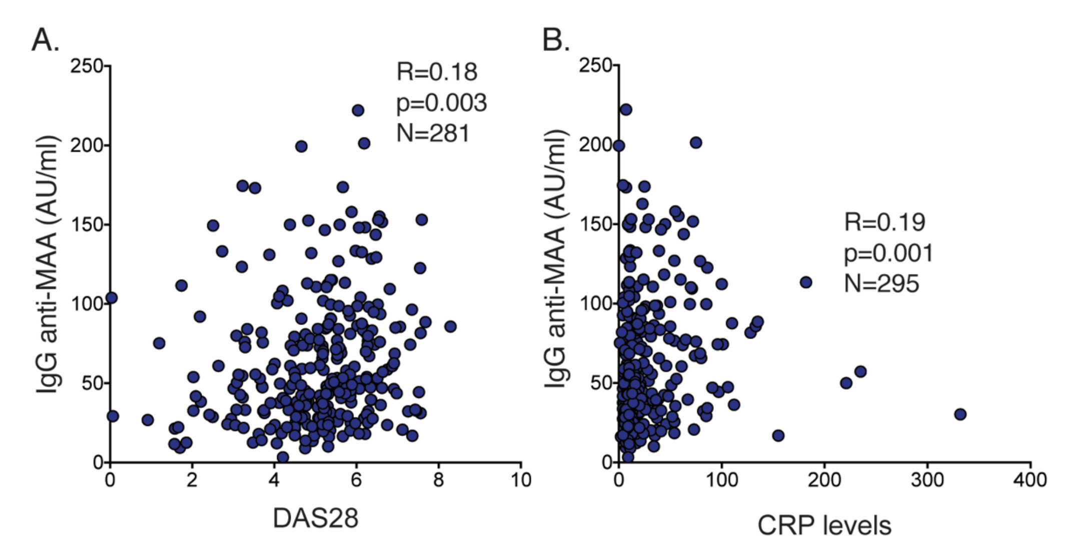

Supplemental Figure 7. Correlation between IgG anti-MAA levels and RA disease activity and CRP levels

IgG anti-MAA levels by ELISA in RA patients in association with DAS28 disease activity (**A**) and CRP (**B**). P-values and R-values from Spearman correlation.

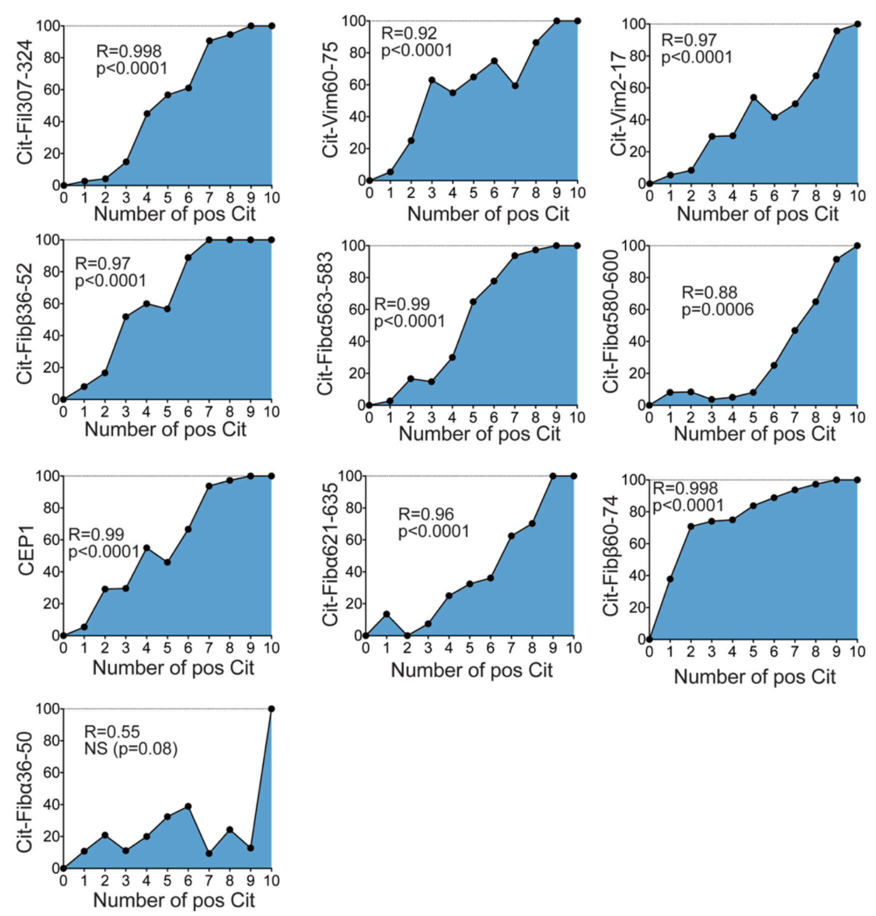

Supplemental Figure 8. Correlation of ACPA fine-specificity with the number of positive Cit-peptide reactivities

Correlation of the frequency of individual Cit-reactivities with the total number of positive ACPA fine-specificities in 402 RA patients. R-values and p-values are presented from Spearman correlation.

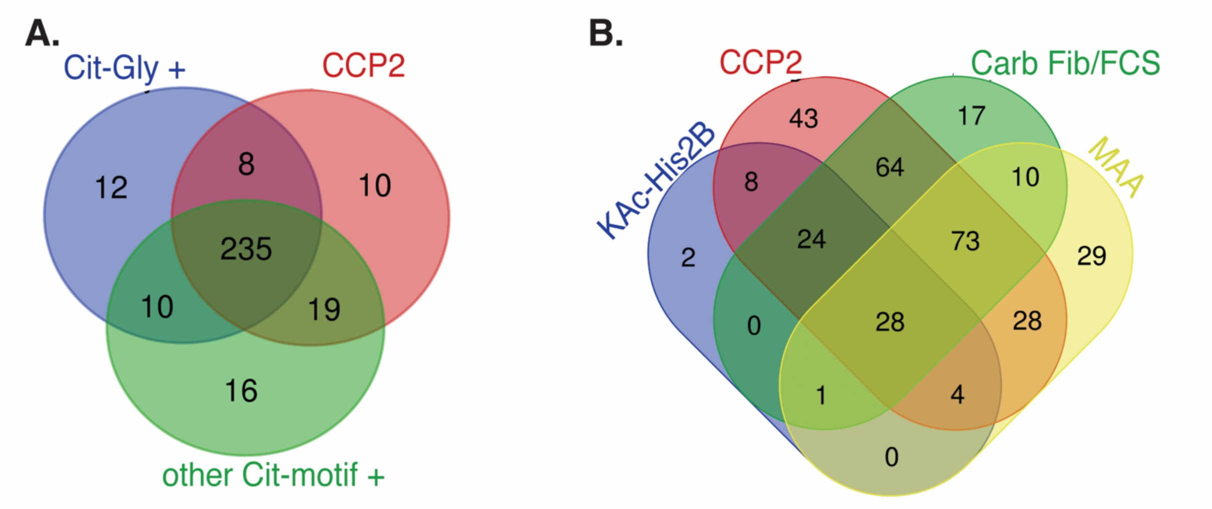

Supplemental Figure 9. Association between different AMPA reactivities in RA

Data is shown from 386 patients with complete citrulline data comparing CCP2 IgG (CCPlus Euro Diagnostica), citrulline peptide reactivity by antigen microarray (Thermo Fisher Scientific, 10 Cit-peptides included, see Supplemental Table 1), ELISA IgG anti-KAc-His2B(K12), any Carb reactivity (IgG anti-Carb-Fibrinogen and/or IgG anti-Carb-FCS), and IgG anti-MAA-BSA. Venn diagrams were generated using an online tool (http://bioinformatics.psb.ugent.be/webtools/Venn/).

A.

% pos. of	IgG	Cit +	Orn(Ac) +	Carb +	Lys(Ac) +
Cit +	*(n=84)*		58,0%	36,0%	20,2%
Orn(Ac) +	*(n=56)*	87,5%		46.4%	28,6%
Carb +	*(n=44)*	68,2%	59,1%		47.7%
Lys(Ac) +	*(n=32)*	53,0%	50,0%	65,6%	

B.

**Frequencies**	**Controls**	**CCP2 neg & RF IgM neg subset (with AMPA screen n=70)**
Δac-lys His2B IgG pos	0% (0/437)	0% (0/70)
Mod-Vim IgG Cit pos	2.9% (14/480)	8.6% (6/69)
Mod-Vim IgG Orn(Ac) pos	2.7% (13/480)	4.3% (3/69)
Mod-Vim IgG Carb pos	2.7% (13/480)	4.3% (3/69)
Mod-Vim IgG Lys(Ac) pos	2.9% (14/480)	4.3% (3/69)
Mod-Vim. IgA Cit pos	0.6% (3/480)	0% (0/69
Mod-Vim IgA Orn(Ac) pos	0.6% (3/480)	0% (0/69
Mod-Vim IgA Carb pos	0% (0/480)	1.4% (1/69)
Mod-Vim IgA Lys(Ac) pos	0.2% (1/480)	1.4% (1/69)
Carb-Fib IgG pos	2.5% (8/316)	22% (15/69)
Carb-FCS IgG pos	3.1% (10/316)	8.6% (6/69)
MAA IgG high	14% (61/437)	36% (25/70)
RF IgG pos	6.0% (37/625)	8.6% (6/70)
RF IgA pos	1.3% (8/625)	0% (0/69)

C.

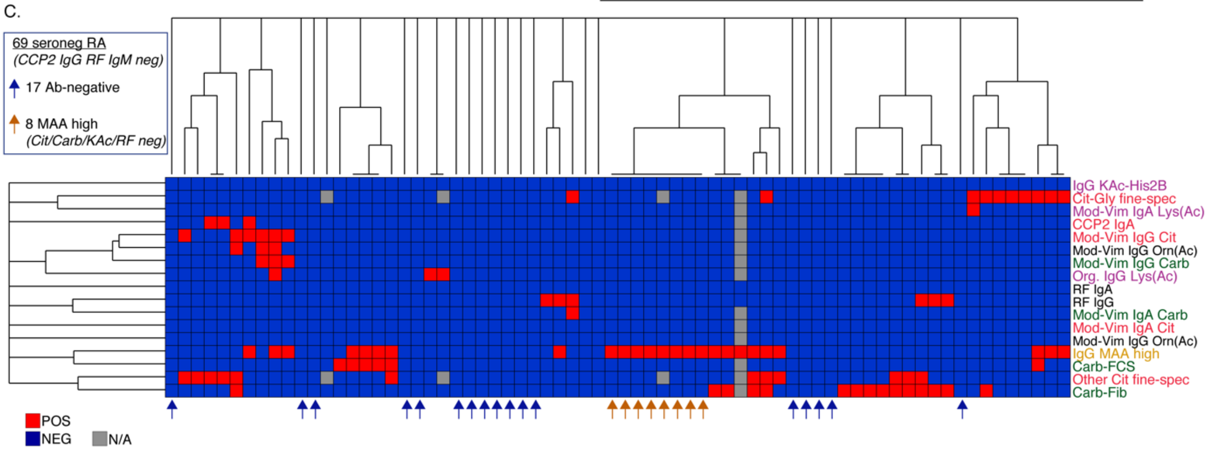

Supplemental Figure 10. Autoantibody reactivities in "seronegative" RA

A. Association between mod-Vim IgG reactivities in 733 CCP2 negative RA patients. **B-C.** 70 of 124 CCP2 negative patients included in the IgG anti-KAc-His2B and MAA screen, were negative for both CCP2 IgG and RF IgM. Distribution of other autoreactivities in these patients showed that only 17 were negative in all Cit/Carb/KAc/RF tests as well as the high anti-MAA IgG test (three had incomplete tests).

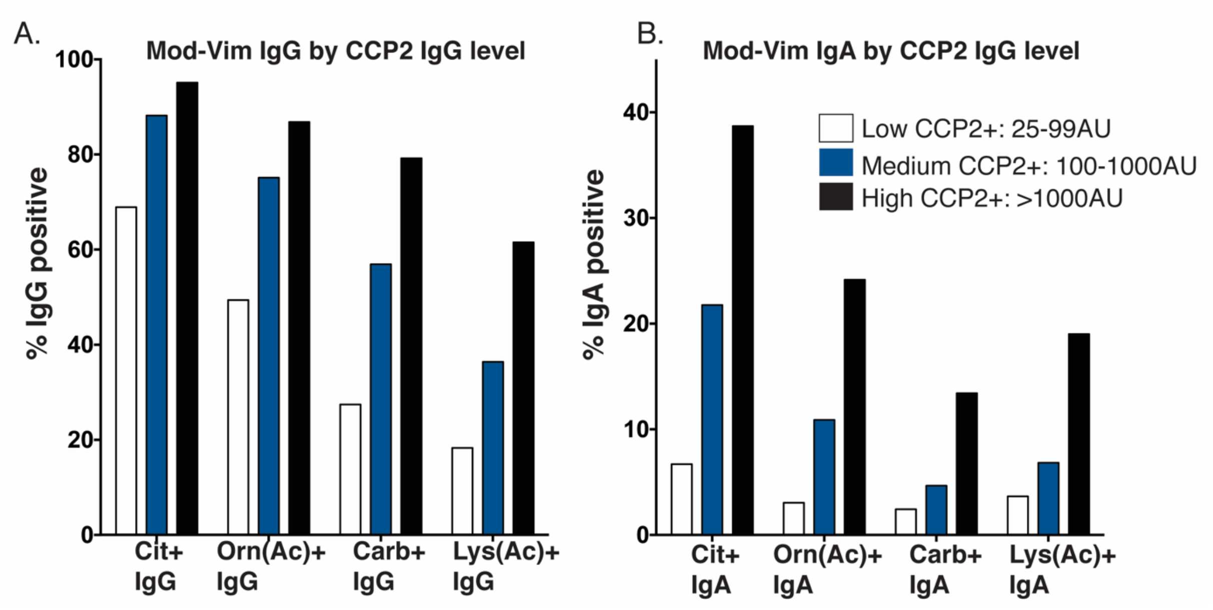

Supplemental Figure 11. Association of IgG AMPA and IgA AMPA reactivity with high CCP2 IgG levels.

CCP2+ RA patients (N=1259) were dichotomized based the anti-CCP2 levels: low (25-99AU), medium (100-1000AU) or high (>1000 AU), and the frequency of IgG (**A**) and IgA (**B**) positivity in the modified-vimentin assays were compared. The Euro Diagnostica CCPlus assay has a cutoff for positivity at 25 AU and saturates at 3200 AU.

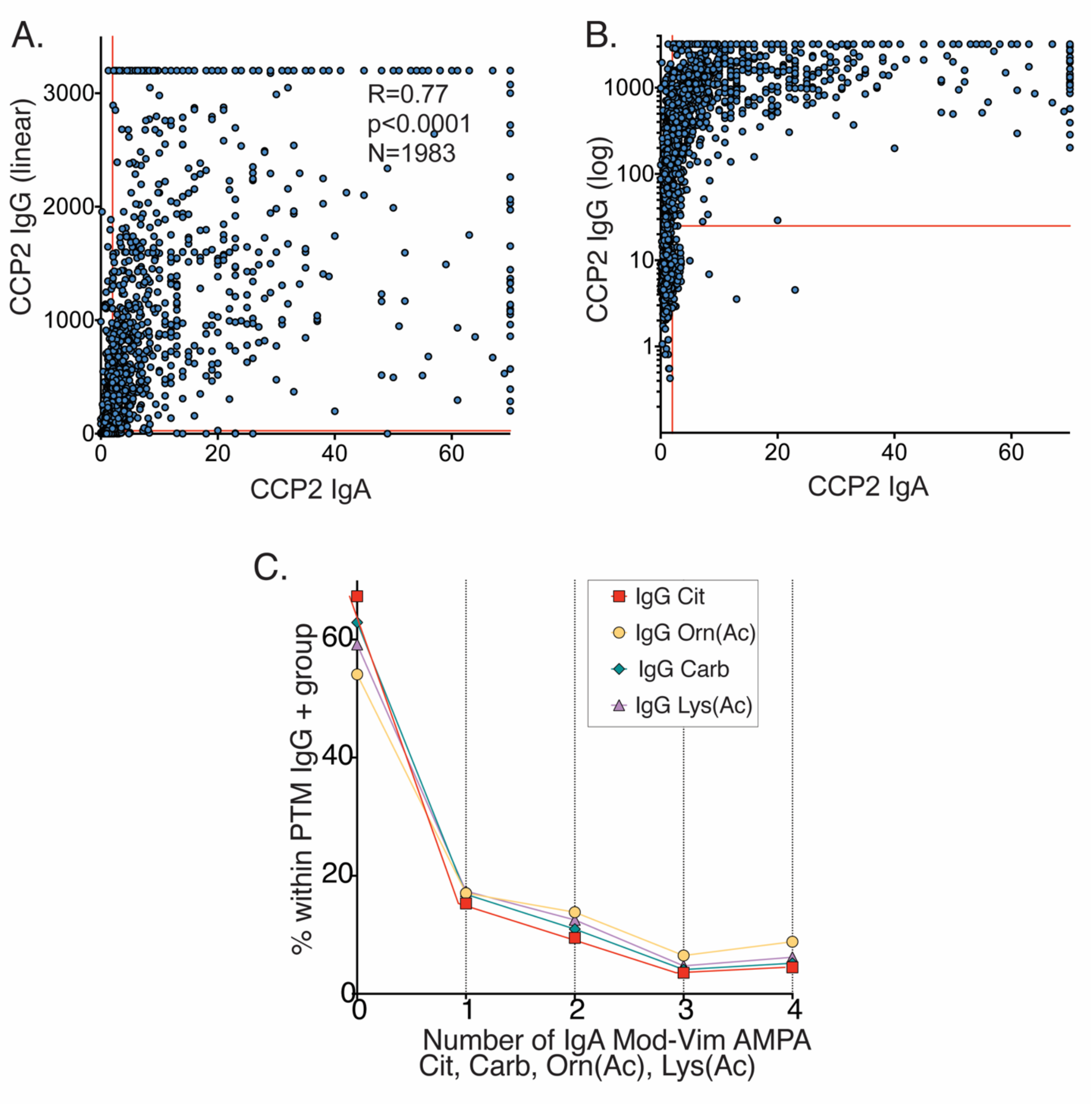

Supplemental Figure 12. IgA AMPA reactivity in association to IgG CCP2 levels and number of different AMPA reactivities

Panel (**A-B**) show the association between CCP2 IgG serum levels and CCP2 IgA serum levels either using a linear or a logarithmic scale. **C**. The graph depicts the frequency of multiple IgA AMPA positive tests by the mod-Vim assay (IgA anti-Cit, anti-Carb, anti-Orn(Ac), anti-Lys(Ac)) within the different IgG+ groups. There was not a higher probability of multiple IgA AMPA in any of the PTM IgG+ subsets.

Supplemental Table 1. Peptides used in RA screening

Peptide	Assay	Sequence	Ref
Cit-peptides with cit-gly motif			
Cit-Fil$_{307-324}$ (cyclic)	antigen-array*	---------------SHQEST**(Cit)**GRSRGRSGRSGS	[7]
CEP-1 (cyclic)	antigen-array	-------KIHA**(Cit)**EIFDS**(Cit)**GNPTVE	[8]
Cit-Fibβ$_{36-52}$	antigen-array	-------------NEEGFFSA**(Cit)**GHRPLDKK	[9]
Cit-Fibα$_{563-583}$	antigen-array	-----------HHPGIAEFPS**(Cit)**GKSSSYSKQF	[10]
Cit-Fibα$_{580-600}$	antigen-array	----------SKQFTSSTSYN**(Cit)**GDSTFESKS	[11]
Cit-Fibα$_{621-635}$	antigen-array	----**(Cit)**GHAKS**(Cit)**PV**(Cit)**GIHTS	[12, 13]
Cit-peptides with other motifs			
Cit-Vim$_{60-75}$	antigen-array	-VYAT**(Cit)**SSAV**(Cit)**L**(Cit)**SSVP	[9]
Cit-Vim$_{2-17}$	antigen-array	-ST**(Cit)**SVSSSSY**(Cit)(Cit)**MFGG	[14]
Cit-Fibα$_{36-50}$	antigen-array	GP**(Cit)**VVE**(Cit)**HQSASKDS	[12, 13]
Cit-Fibβ$_{60-74}$	antigen-array	**(Cit)**PAPPPISGGGY**(Cit)**A**(Cit)**	[12, 13]
KAc-peptides			
His2B_AcK12 (cyclic)	ELISA	-----SAPAPK**(KAc)**GSKKAVTKAQ	[15]
KAc-His4_1-18	ELISA	SGRG**(KAc)**GG**(KAc)**GLG**(KAc)**GGA**(KAc)**RH	[15]
KAc5-His4	ELISA	---SGRG**(KAc)**GGKGLGKGGAKRH	[15]
KAc16-His4	ELISA	------SGRGKGGKGLGKGGA**(KAc)**RH	
Carb-peptides			
Fibβ$_{43-56}$ Carb	ELISA	ARGHRPLDK**(Carb)**REEA	[3]
Fibβ$_{77-87}$ Carb (cyclic)	ELISA	AKAAATQ**(Carb)**KVER	[3]
Carb-CEP-1 (cyclic)	antigen-array	KIHA**(Carb)**EIFDS**(Carb)**GNPTVE	[16]
mod-Vim assays			
Cit ModVim$_{58-69}$	ELISA	GRVYAT**(Cit)**SSAVR	[17]
Carb ModVim$_{58-69}$	ELISA	GRVYAT**(Carb)**SSAVR	
Lys(Ac) ModVim$_{58-69}$	ELISA	GRVYAT**(KAc)**SSAVR	
Carb Orn(Ac) ModVim$_{58-69}$	ELISA	GRVYAT**(OrnAc)**SSAVR	

* The ACPA fine-specificity array is further described in [18, 19]. Glycine in position +1 or -1 compared to the modified residue is underlined.

Supplemental Table 2. Screening of control assays

	Controls	SLE	RA-Risk	All RA	CCP-RA	CCP+ RA
Control assays						
Lys-His2B IgG (AU/ml)	1.5±3.3 (0.56)	6.45±10 (1.9)	3.0±5.8 (1.0)	1.3±2.4 (0.6)	0.93±1.6 (0.45)	1.4±2.7 (0.67)
mod-Vim Arg IgG (OD)	0.10±0.20 (0.09)	N/A	N/A	0.14±0.12 (0.13)	0.14±0.13 (0.12)	0.14±0.12 (0.13)
mod-Vim Lys IgG OD)	0.11±0.06 (0.10)	N/A	N/A	0.17±012 (0.14)	0.15±0.08 (0.14)	0.17±0.14 (0.15)
mod-Vim Orn IgG (OD)	0.16±0.20 (0.12)	N/A	N/A	0.19±0.15 (0.16)	0.17±0.12 (0.14)	0.20±0.17 (0.17)
mod-Vim Arg IgA (OD)	0.05±0.02 (0.05)	N/A	N/A	0.08±0.05 (0.07)	0.08±0.07 (0.07)	0.07±0.04 (0.07)
mod-Vim Lys IgA (OD)	0.06±0.01 (0.05)	N/A	N/A	0.09±0.05 (0.08)	0.10±0.06 (0.10)	0.09±0.05 (0.08)
mod-Vim Orn IgA (OD)	0.07±0.07 (0.05)	N/A	N/A	0.10+±0.06 (0.09)	0.10±0.08 (0.09)	0.10±0.05 (0.08)

His2B assays were screened in 437 Population controls, 160 SLE patients, 267 RA-risk, 403 RA patients (whereof 278 CCP2+)
Mod-Vim assays were screened in 480 Population controls, 1984 RA patients (whereof 1249 CCP2+)

Supplementary Table 3. AMPA associations with smoking in CCP2+ RA

	**ACPA+ no smoking**	**ACPA+ with ever smoking#**	*OR [CI]*	*p-value**	*Adjusted p-value***
AMPA IgG anti-					
CCP2 levels (Mean± SD [Median; N)	903±967 [525; N=489]	1060±996 [700; N=759]		**0.0004**	
Δac-lys His2B pos [>4.25 AU/ml]*	12.5% (9/72)	27.3 % (56/205)	2.6 [1.2-5.6]	**0.01**	NS (p=0.10)
mod-Vim Cit pos [>0.55 AU/ml]	87% (425/489)	89% (678/759)	1.3 [0.89-1.8]	NS (p=0.20)	
mod-Vim Orn(Ac) pos [OD>0.55]#	74% (362/489)	77% (588/759)	1.1 [0.92-1.6]	NS (p=0.20)	
mod-Vim Carb pos [OD>0.69]#	59% (288/489)	63% (476/759)	1.2 [0.93-1.5]	NS (p=0.20)	
mod-Vim Lys(Ac) pos [OD>0.68]#	43% (208/489)	46% (330/759)	1.0 [0.82-1.3]	NS (p=0.82)	
Carb-Fib pos [>23 AU/ml]§	57% (280/489)	62% (473/759)	1.2 [0.97-1.5]	NS (p=0.09)	NS (0.27)
Carb-FCS pos [>232 AU/ml]§	44% (216/489)	53% (403/759)	1.4 [1.1-1.8]	**0.002**	**0.01**
MAA high [>56.44 AU/ml]**	63% (45/72)	64% (132/205)	0.99 [0.58-1.7]	NS (p=0.77)	
AMPA IgA anti-					
CCP2 levels	7.7±12 [2.7; N=487]	12.5±17 [4.7; N=758]		**<0.0001**	**<0.0001**
CCP2 pos [>2 AU/ml]	60% (290/487)	75% (569/758)	2.0 [1.6-2.6]	**<0.0001**	**<0.0001**
mod-Vim Cit pos [OD>0.32]€	19% (93/489)	30% (231/759)	1.9 [1.4-2.4]	**<0.0001**	**<0.0001**
mod-Vim Orn(Ac) pos [OD>0.36]#	11% (55/489)	17% (128/759)	1.6 [1.1-2.2]	**0.007**	**0.03**
mod-Vim Carb pos [OD>0.48]#	5% (24/489)	9% (70/759)	2.0 [1.2-3.2]	**0.006**	**0.02**
mod-Vim Lys(Ac) pos [OD>0.36]#	9% (46/489)	12% (89/759)	1.3 [0.9-1.9]	NS (p=0.23)	
Total IgA levels	2.3±0.96 [2.2; N=485]	2.4± 0.97 [2.3; N=755]		**0.02**	NS (0.16)
Rheumatoid factor					
RF IgM pos	88% (431/489)	92% (698/759)	1.5 [1.1-2.2]	**0.03**	**0.04**
RF IgG pos	73% (361/489)	82% (626/759)	1.7 [1.3-1.2]	**0.0003**	**0.001**
RF IgA pos	47% (234/489)	67% (510/759)	2.2 [1.8-2.8]	**<0.0001**	**<0.0001**

*p-value from Fisher's exact test or Mann-Whitney analysis
** p-value from logistic regression analysis adjusting for CCP2 IgG levels
# Either previous or current cigarette smoking

Supplementary Table 4. AMPA associations HLA shared epitope

	All RA patients				*CCP2 IgG+ RA patients*			
	SE-	*SE+*	*OR [CI]*	*p-value**	*SE-*	*SE+*	*OR [CI]*	*p-value**
AMPA IgG anti-								
ΔAc-His2B$_{6\text{-}22}$ pos	13% (92/92)	18% (55/305)	1.5 [0.75-2.9]	NS (p=0.34)	32% (12/37)	22% (52/239)	0.58 [0.27-1.2]	NS (p=0.21)
Carb-CEP1 pos	15% (13/89)	32% (96/299)	2.8 [1.5-5.2]	**0.001**	31% (11/35)	38% (90/235)	1.4 [0.63-2.9]	NS (p=0.46)
mod-Vim Cit pos	20% (50/245)	54% (272/507)	4.5 [3.2-6.4]	**<0.0001**	73% (37/51)	89% (255/287)	3.0 [1.5-6.2]	**0.004**
mod-Vim Orn(Ac) pos	17% (41/245)	46% (232/507)	4.2 [2.9-6.2]	**<0.0001**	59% (30/51)	76% (219/287)	2.3 [1.2-4.2]	**0.01**
mod-Vim Carb pos	13% (31/245)	34% (173/507)	3.6 [2.4-5.4]	**<0.0001**	37% (19/51)	57% (163/287)	2.2 [1.2-4.1]	**0.01**
mod-Vim Lys(Ac) pos	11% (27/245)	28% (143/507)	3.2 [2.0-4.9]	**<0.0001**	35% (18/51)	47% (134/287)	1.6 [0.87-3.0]	NS (p=0.17)
Carb-Fib pos	26% (65/245)	51% (261/507)	2.9 [2.1-4.1]	**<0.0001**	63% (32/51)	76% (218/287)	1.9 [1.0-3.6]	NS (p=0.06)
Carb-FCS pos	21% (53/245)	40% (205/507)	2.5 [1.7-3.5)	**<0.0001**	52% (27/51)	62% (179/287)	1.5 [0.81-2.7]	NS (p=0.22)
MAA high	42% (39/92)	44% (133/305)	1.1 [0.66-1.7]	NS (p=0.90)	51% (19/37)	48% (115/239)	0.88 [0.44-1.8]	NS (0.72)
AMPA IgA anti-								
CCP2 pos	23% (56/245)	42% (212/508)	2.4 [1.7-3.5]	**<0.0001**	75% (38/51)	69% (200/288)	0.78 [0.39-1.5]	NS (p=0.51)
mod-Vim Cit pos	8% (20/245)	21% (106/507)	3.0 [1.8-5.0]	**<0.0001**	35% (18/51)	36% (103/287)	1.0 [0.56-1.9]	NS (p>0.99)
mod-Vim Orn(Ac) pos	7% (18/245)	16% (83/507)	2.5 [1.4-4.2]	**0.0006**	25% (13/51)	27% (76/287)	1.1 [0.55-2.1]	NS (p>0.99)
mod-Vim Carb pos	7% (18/245)	7% (37/507)	0.99 [0.55-1.8]	NS (p>0.99)	16% (8/51)	11% (31/287)	0.65 [0.28-1.5]	NS (p=0.33)
mod-Vim Lys(Ac) pos	7% (17/245)	9% (45/507)	1.3 [0.73-2.3]	NS (p=0.40)	25% (13/51)	15% (43/287)	0.51 [0.25-1.0]	NS (p=0.07)
RF								
RF IgG pos	30% (75/247)	52% (265/507)	2.5 [1.8-3.4]	**<0.0001**	94% (48/51)	81% (235/287)	0.27 [0.08-0.91]	**0.02**
RF IgA pos	22% (55/247)	40% (202/507)	2.3 [1.6-3.2]	**<0.0001**	75% (38/51)	64% (184/287)	0.60 [0.31-1.2]	NS (p=0.15)
RF IgM pos	31% (79/247)	60% (307/507)	3.2 [2.3-4.4]	**<0.0001**	92% (47/51)	91% (264/287)	0.90 [.030-2.7]	NS (p>0.99)

SE: HLA-DRB1 shared epitope
* p-value from Fisher's exact test
OR= Odds ratio, CI= 95% confidence interval

Supplemental Table 5. Inhibitory effect *HLA DRB1*03* on AMPA expression in CCP2+ shared epitope positive RA

	***HLA DRB1*03* negative**	***HLA DRB1*03* positive**	*OR [CI]*	*p-value**	*Adjusted p-value***
IgG CCP2 high (>1000)	40% (186/460)	27% (24/88)	0.55 [0.30-1.0]	**p=0.05**	
AMPA IgG anti-					
ΔAc-His2B$_{6-22}$ pos	24% (50/208)	6.5% (2/31)	0.22 [0.05-0.95]	**p=0.03**	NS (0.11)
Carb-CEP1 pos	40% (82/204)	26% (8/31)	0.52 [0.22-1.21]	NS (p=0.17)	
mod-Vim Cit pos	89% (219/246)	88% (36/41)	0.9 [0.32-2.5]	NS (p=0.79)	
mod-Vim Orn(Ac) pos	77% (190/246)	71% (29/41)	0.73 [0.35-1.52]	NS (p=0.43)	
mod-Vim Carb pos	57% (141/246)	55% (22/41)	0.89 [0.46-1.72]	NS (p=0.74)	
mod-Vim Lys(Ac) pos	49% (121/246)	31% (13/41)	0.48 [0.23-0.97]	**0.04**	NS (0.10)
Carb-Fib pos	77% (189/246)	74% (31/41)	0.86 [0.41-1.8]	NS (p=0.70)	
Carb-FCS pos	65% (161/246)	47% (19/41)	0.44 [0.22-0.86]	**0.02**	**0.04**
MAA high	48% (100/208)	48% (15/31)	1.0 [0.48-2.2]	NS (p>0.99)	
AMPA IgA anti-					
CCP2 pos	70% (173/245)	61% (25/41)	0.6 [0.32-1.3]	NS (p=0.27)	
mod-Vim Cit pos	38% (94/246)	23% (9/41)	0.45 [0.21-0.96]	NS (p=0.053)	
mod-Vim Orn(Ac) pos	28% (67/246)	24% (9/41)	0.75 [0.34-1.7]	NS (p=0.57)	
mod-Vim Carb pos	12% (30/246)	2.4% (1/41)	0.18 [0.02-1.34]	NS (p=0.10)	
mod-Vim Lys(Ac) pos	17% (41/246)	4.8% (2/41)	0.25 [0.06-1.1]	NS (p=0.06)	
RF					
RF IgG pos	81% (200/246)	80% (33/41)	0.95 [0.41-2.2]	NS (p=0.83)	
RF IgA pos	65% (160/246)	58% (24/41)	0.76 [0.38-1.5]	NS (p=0.48)	
RF IgM pos	90% (223/246)	95% (39/41)	2.0 [0.46-8.7]	NS (p=0.54)	

The analysis included CCP2+ RA patients with HLA-DR shared epitope positivity
*p-value from Fisher's exact test or Mann-Whitney analysis
** p-value from logistic regression analysis adjusting for CCP2 IgG levels
OR= Odds ratio, CI= 95% confidence interval

Supplemental Table 6. IgG anti-MAA correlates with inflammatory components of the DAS28 disease activity score

	**R-value**	**p-value***
DAS28	0.15	**0.02**
DAS28_CRP	0.18	**0.03**
CRP baseline	0.15	**0.007**
ESR	0.18	**0.001**
SJC	0.14	**0.01**
TJC	0.08	0.16
Pain	-0.0009	0.87
Patient global	0.015	0.79
HAQ	0.009	0.87

*p-values from Spearman analysis

Supplemental Table 7. Correlation of IgG anti-MAA with 6-month follow up of disease activity

	**median IgG anti-MAA low (≤43.4)**	**median IgG anti-MAA interm & high (>43.4)**	**p-value***	**median IgG anti-MAA low (≤56.44)**	**median IgG anti-MAA high (>56.44)**	**p-value***
Δ 6 months CRP	-5,5	-10	0.050	-6	-10	0.097
Δ 6 months ESR	-8	-14	**0.004**	-10	-14	0.068
Δ 6 months SJC	-5	-6	0.31	-6	-6	0.72
Δ 6 months TJC	-3.5	-5	0.29	-4	-5.5	0.35
Δ 6 months pain VAS	-19.5	-21	0.72	-19.5	-21	0.72
Δ 6 months global VAS	-17.5	-17	0.72	-19	-16	0.93
Δ 6 months HAQ	-0.38	-0.37	0.71	-0.38	-0.37	0.81
Δ 6 months DAS28	-1.72	-1.92	0.30	-1.79	-1.92	0.41
Δ 6 months DAS28CRP	-1.46	-1.76	0.36	-1.48	-1.86	0.49

*p-values from Spearman analysis

Supplemental Table 8. Frequency of high CCP2 (>1000AU) in AMPA IgG+ RA patients with and without IgA autoantibodies

	**IgG+ IgA-**	**IgG+ IgA+**	*OR [CI]*	*p-value**
CCP2	4.6% (18/388)	50% (427/861)	20 [12-33]	<0.0001
mod-Vim Cit	32% (252/784)	54% (174/324)	2.4 [1.9-3.1]	<0.0001
mod-Vim Orn(Ac)	36% (282/775)	60% (107/179)	2.6 [1.9-3.6]	<0.0001
mod-Vim Carb	44% (298/680)	65% (57/88)	2.4 [1.5-3.7]	0.0002
mod-Vim Lys(Ac)	46% (195/422)	69% (81/118)	2.5 [1.7-3.9]	<0.0001

*p-value from Fisher's exact test
OR= Odds ratio, CI= 95% confidence interval

References

[1] A. Svard, T. Skogh, L. Alfredsson, A. Ilar, L. Klareskog, C. Bengtsson *et al.* Associations with smoking and shared epitope differ between IgA- and IgG-class antibodies to cyclic citrullinated peptides in early rheumatoid arthritis. Arthritis Rheumatol, 2015;67:2032-7.

[2] X. Jiang, L. A. Trouw, T. J. van Wesemael, J. Shi, C. Bengtsson, H. Kallberg *et al.* Anti-CarP antibodies in two large cohorts of patients with rheumatoid arthritis and their relationship to genetic risk factors, cigarette smoking and other autoantibodies. Ann Rheum Dis, 2014;73:1761-8.

[3] J. D. Jones, B. J. Hamilton, W. F. C. Rigby. Brief Report: Anti-Carbamylated Protein Antibodies in Rheumatoid Arthritis Patients Are Reactive With Specific Epitopes of the Human Fibrinogen beta-Chain. Arthritis Rheumatol, 2017;69:1381-6.

[4] H. Bang, K. Egerer, A. Gauliard, K. Luthke, P. E. Rudolph, G. Fredenhagen *et al.* Mutation and citrullination modifies vimentin to a novel autoantigen for rheumatoid arthritis. Arthritis Rheum, 2007;56:2503-11.

[5] M. Juarez, H. Bang, F. Hammar, U. Reimer, B. Dyke, I. Sahbudin *et al.* Identification of novel antiacetylated vimentin antibodies in patients with early inflammatory arthritis. Ann Rheum Dis, 2016;75:1099-107.

[6] U. Hardt, A. Larsson, I. Gunnarsson, R. M. Clancy, M. Petri, J. P. Buyon *et al.* Autoimmune reactivity to malondialdehyde adducts in systemic lupus erythematosus is associated with disease activity and nephritis. Arthritis Res Ther, 2018;20:36.

[7] G. A. Schellekens, H. Visser, B. A. de Jong, F. H. van den Hoogen, J. M. Hazes, F. C. Breedveld *et al.* The diagnostic properties of rheumatoid arthritis antibodies recognizing a cyclic citrullinated peptide. Arthritis Rheum, 2000;43:155-63.

[8] K. Lundberg, A. Kinloch, B. A. Fisher, N. Wegner, R. Wait, P. Charles *et al.* Antibodies to citrullinated alpha-enolase peptide 1 are specific for rheumatoid arthritis and cross-react with bacterial enolase. Arthritis Rheum, 2008;58:3009-19.

[9] K. N. Verpoort, K. Cheung, A. Ioan-Facsinay, A. H. van der Helm-van Mil, J. K. de Vries-Bouwstra, C. F. Allaart *et al.* Fine specificity of the anti-citrullinated protein antibody response is influenced by the shared epitope alleles. Arthritis Rheum, 2007;56:3949-52.

[10] M. Hermansson, K. Artemenko, E. Ossipova, H. Eriksson, J. Lengqvist, D. Makrygiannakis *et al.* MS analysis of rheumatoid arthritic synovial tissue identifies specific citrullination sites on fibrinogen. Proteomics Clin Appl, 2010;4:511-8.

[11] C. Fernandes-Cerqueira, E. Ossipova, S. Gunasekera, M. Hansson, L. Mathsson, A. I. Catrina *et al.* Targeting of anti-citrullinated protein/peptide antibodies in rheumatoid arthritis using peptides mimicking endogenously citrullinated fibrinogen antigens. Arthritis Res Ther, 2015;17:155.

[12] C. Iobagiu, A. Magyar, L. Nogueira, M. Cornillet, M. Sebbag, J. Arnaud *et al.* The antigen specificity of the rheumatoid arthritis-associated ACPA directed to citrullinated fibrin is very closely restricted. J Autoimmun, 2011;37:263-72.

[13] M. Sebbag, N. Moinard, I. Auger, C. Clavel, J. Arnaud, L. Nogueira *et al.* Epitopes of human fibrin recognized by the rheumatoid arthritis-specific autoantibodies to citrullinated proteins. Eur J Immunol, 2006;36:2250-63.

[14] O. Snir, M. Widhe, M. Hermansson, C. von Spee, J. Lindberg, S. Hensen *et al.* Antibodies to several citrullinated antigens are enriched in the joints of rheumatoid arthritis patients. Arthritis Rheum, 2010;62:44-52.

[15] K. A. Lloyd, G. Wigerblad, P. Sahlström, M. G. Garimella, K. Chemin, J. Steen *et al.* Differential ACPA Binding to Nuclear Antigens Reveals a PAD-Independent Pathway and a Distinct Subset of Acetylation Cross-Reactive Autoantibodies in Rheumatoid Arthritis. Front Immunol, 2019;9:3033.

[16] E. Reed, X. Jiang, N. Kharlamova, A. J. Ytterberg, A. I. Catrina, L. Israelsson *et al.* Antibodies to carbamylated alpha-enolase epitopes in rheumatoid arthritis also bind citrullinated epitopes and are largely indistinct from anti-citrullinated protein antibodies. Arthritis Res Ther, 2016;18:96.

[17] C. P. Figueiredo, H. Bang, J. F. Cobra, M. Englbrecht, A. J. Hueber, J. Haschka *et al*. Antimodified protein antibody response pattern influences the risk for disease relapse in patients with rheumatoid arthritis tapering disease modifying antirheumatic drugs. Ann Rheum Dis, 2017;76**:**399-407.

[18] M. Hansson, L. Mathsson, T. Schlederer, L. Israelsson, P. Matsson, L. Nogueira *et al*. Validation of a multiplex chip-based assay for the detection of autoantibodies against citrullinated peptides. Arthritis Res Ther, 2012;14**:**R201.

[19] J. Ronnelid, M. Hansson, L. Mathsson-Alm, M. Cornillet, E. Reed, P. J. Jakobsson *et al*. Anticitrullinated protein/peptide antibody multiplexing defines an extended group of ACPA-positive rheumatoid arthritis patients with distinct genetic and environmental determinants. Ann Rheum Dis, 2018;77**:**203-11.
